# Murine trophoblast-derived and pregnancy-associated exosome-enriched extracellular vesicle microRNAs: Implications for placenta driven effects on maternal physiology

**DOI:** 10.1371/journal.pone.0210675

**Published:** 2019-02-07

**Authors:** Adrianne L. Stefanski, Nadine Martinez, Lisa K. Peterson, Tiffany J. Callahan, Eric Treacy, Marisa Luck, Samantha F. Friend, Amy Hermesch, Emin Maltepe, Tzu Phang, Leonard L. Dragone, Virginia D. Winn

**Affiliations:** 1 Department of Obstetrics and Gynecology, University of Colorado School of Medicine, Aurora, CO, United States of America; 2 Department of Medicine, Division of Pulmonary Sciences and Critical Care Medicine, University of Colorado School of Medicine, Aurora CO, United States of America; 3 Department of Obstetrics and Gynecology, Stanford University School of Medicine, Stanford, CA, United States of America; 4 Department of Pediatrics, National Jewish Health, Denver CO, United States of America; 5 Computational Biosciences Program, University of Colorado School of Medicine, Aurora, CO, United States of America; 6 Department of Medicine, University of Colorado School of Medicine, Aurora CO, United States of America; 7 Department of Pediatrics, University of Colorado School of Medicine, Aurora CO, United States of America; Academic Medical Centre, University of Amsterdam, NETHERLANDS

## Abstract

The role of extracellular vesicles (EVs), specifically exosomes, in intercellular communication likely plays a key role in placental orchestration of pregnancy and maternal immune sensing of the fetus. While murine models are powerful tools to study pregnancy and maternal-fetal immune interactions, in contrast to human placental exosomes, the content of murine placental and pregnancy exosomes remains largely understudied. Using a recently developed *in vitro* culture technique, murine trophoblast stem cells derived from B6 mice were differentiated into syncytial-like cells. EVs from the conditioned media, as well as from pregnant and non-pregnant sera, were enriched for exosomes. The RNA composition of these murine trophoblast-derived and pregnancy-associated exosome-enriched-EVs (ExoE-EVs) was determined using RNA-sequencing analysis and expression levels confirmed by qRT-PCR. Differentially abundant miRNAs were detected in syncytial differentiated ExoE-EVs, particularly from the X chromosome cluster (mmu-miR-322-3p, mmu-miR-322-5p, mmu-miR-503-5p, mmu-miR-542-3p, and mmu-miR-450a-5p). These were confirmed to be increased in pregnant mouse sera ExoE-EVs by qRT-PCR analysis. Interestingly, fifteen miRNAs were only present within the pregnancy-derived ExoE-EVs compared to non-pregnant controls. Mmu-miR-292-3p and mmu-miR-183-5p were noted to be some of the most abundant miRNAs in syncytial ExoE-EVs and were also present at higher levels in pregnant versus non-pregnant sera ExoE-EVs. The bioinformatics tool, MultiMir, was employed to query publicly available databases of predicted miRNA-target interactions. This analysis reveals that the X-chromosome miRNAs are predicted to target ubiquitin-mediated proteolysis and intracellular signaling pathways. Knowing the cargo of placental and pregnancy-specific ExoE-EVs as well as the predicted biological targets informs studies using murine models to examine not only maternal-fetal immune interactions but also the physiologic consequences of placental-maternal communication.

## Introduction

Appreciation of intercellular communication has shifted dramatically since the identification and demonstrated role of extracellular vesicles (EVs). EVs are released from cells, carrying protein and nucleic acids that then impact function(s) of distant cells and tissues. The human placenta, a major regulator of maternal physiology during pregnancy, is a prime example of this type of cellular communication. The outer syncytiotrophoblast layer of the chorionic villi of human placentas is bathed in maternal blood and releases a wide range of EVs including exosomes, microvesicles, apoptotic bodies and large cellular fragments including syncytial nuclear aggregates [1]. Pregnancy causes a 50-fold increase in circulating exosomes, and placenta-derived exosomes are known to be present in maternal blood [2, 3]. In general, exosomes are thought to be anti-inflammatory or tolerogenic upon uptake into recipient cells [2]. Therefore, placental exosomes may play an important role in maternal-fetal tolerance.

Exosomes contain distinct repertoires of proteins, lipids, and RNAs originating from the host cell. Placental EVs carry a wide range of proteins with known or predicted functional effects on target cells including the pro-apoptotic protein members of the tumor necrosis factor (TNF) family, FAS ligand and TNF-related apoptosis inducing ligand (TRAIL) [4–6]. Human placental exosomes are known to express regulatory molecules including programmed death-ligand 1 (PDL-1) and the cytokine transforming growth factor β (TGF- β) [7]. These proteins are capable of altering immunological responses in target cells [7]. However, less is known about the potential for the RNA-mediated functional alterations in target cells.

Exosomes can transfer RNA to target cells where messenger RNA (mRNA) can be translated into protein and microRNAs (miRNAs) can alter recipient cell gene expression [8]. The abundance of miRNAs within exosomes appears to be non-random, with preferential enrichment for subsets of the miRNAs present in the cell from which the exosome was derived [9]. Sorting of miRNA into exosomes may be a highly regulated process, which has not been fully characterized. Classification of miRNAs expressed in human placentas reveal human-specific clusters [10]. The content of human placental-derived exosome miRNAs have also been reported [7]. Less is known about the miRNA composition in the murine placenta. Recently, global sequencing identified miRNAs in placental tissue, some of which were highly expressed, including two X chromosome cluster miRNAs: mmu-mir-322 and mmu-mir-503 [11]. However, the miRNA content of murine placental extracellular vesicles and particularly exosomes has not previously been reported.

Mice are often used to model normal and pathologic pregnancy, including studies investigating the biological mechanisms responsible for maternal-fetal tolerance in normal pregnancy and systemic alterations in disease. Determining the RNA cargo of placental exosomes is an important first step in understanding their role in pregnancy. We postulate RNAs that are enriched in or unique to pregnancy sera exosomes, and also present in trophoblast-derived exosomes, will reflect placenta-derived RNAs. Therefore, this study sought to characterize the exosomal RNA composition of both *in vitro*-derived murine trophoblastic exosomes and pregnancy-associated exosomes. A recently described *in vitro* model of mouse trophoblast syncytial differentiation from undifferentiated trophoblast stem cells (TSC) was used to generate both stem cell and syncytial derived trophoblast (SynT) EVs [12]. To gain extensive and detailed knowledge about the RNA cargo, next-generation RNA sequencing (RNA Seq) was used. Given the known role of miRNAs of altering recipient cell gene expression, this investigation focused on the miRNA content. Further, analysis of predicted targets of the miRNAs using MultiMir R package was performed. Determining the potential targets of miRNAs contained within trophoblast-derived and pregnancy-specific exosome enriched extracellular vesicles (ExoE-EVs) will inform studies examining the functional alterations anticipated upon placental exosome acquisition by maternal cells.

## Materials and methods

### Cell culture

Murine TSCs derived from C57BL/6 mice were maintained on CellStart-CTS coated plastic plates in RPMI-1640 with L-glutamine (Hyclone SH30027) supplemented with 1% penicillin/streptomycin (Hyclone SV30010), 1% sodium pyruvate (Gibco 11360), 85.8 μM β-mercaptoethanol (Bio-Rad 161–0710), 1 μg/ml heparin (Sigma H3149), 25 ng/ml fibroblast growth factor 4 (FGF4) (Sigma F8424) to maintain stemness. TSCs cultured on plastic in the absence of FGF4 or heparin at normal oxygen tension (O_2_, 21%) result in fused, multinuclear syncytial-like cultures (SynT) [12]. Cell differentiation was monitored by morphology and the down regulation of stem markers CDX2, EOMES, and ELF5 (S1 Fig). The culture media was supplemented with 20% exosome-free fetal bovine serum (System Biosciences EXO-FBS) for RNA sequencing samples or with 20% fetal bovine serum (Hyclone SH30071) depleted of exosomes by ultracentrifugation at 100,000 x g for 16 hours for the immunoblot and qRT-PCR samples.

### Mice

C57BL/6.J (B6) mice (JAX stock #000664) were purchased or bred and housed in a pathogen-free animal care facility at National Jewish Health (NJH) in strict accordance with the recommendations in the Guide for the Care and Use of Laboratory Animals of the National Institutes of Health. The protocol was approved by the Committee on the Ethics of Animal Experiments at NJH. Mice were given ad lib access to food and water. All cages contained bedding and were on 12 hour light-day cycles. At 6–8 weeks of age, B6 mice were pairbred overnight. The presence of a vaginal plug marked gestational day (GD) 0.5. Pregnant female mice were sacrificed on GD 14.5. Age-matched, non-pregnant females (NP) were sacrificed in conjunction with each pregnant female. At time of sacrifice (by carbon dioxide asphyxiation followed by bilateral pneumothorax), blood was collected via cardiac puncture. Serum was prepared from the whole blood using venous blood collection serum separator tubes (BD Vacutainer #367986) per manufacturer’s instructions. Serum was stored at -80°C.

### Isolation of exosome-enriched extracellular vesicles

Conditioned media (CM) was collected from independent TSC (n = 3–5) and SynT (n = 3–5) cultures. Cellular debris was removed by sterile filtering (0.22 μm, Millipore) as described in the alternate filtration protocol by Théry *et al* [13]. ExoE-EVs were isolated from the filtered-CM (10 ml) using ExoQuick-TC (System Biosciences) per manufacturer’s instructions. ExoE-EVs were isolated from NP (n = 11) and GD 14.5 (n = 11) serum (250–500 μl) using ExoQuick (System Biosciences) per manufacturer’s instructions.

### Electron microscopy

ExoE-EVs (10uL) were deposited on Formvar carbon coated, glow-discharged grids using a Denton Desk III TSC Sputter Coater (Electron Microscopy Sciences, Cat. No. FF300-Cu). After 20 minutes of adsorption, grids were fixed with 4% PFA in NaCac for 10 minutes. Grids were then quenched with 50 mM of Glycine in PBS for five minutes then washed with PBS x 3 in 50 uL for 5 minutes each wash. The grids were incubated in blocking media containing 1% BSA in PBS for 20 minutes. After washing with PBS as indicated above, EVs were exposed to either anti-CD63 (LifeTechnologies, Cat. No. 10628D) or anti-Tsg101 (Santa Cruz, sc-7964) for 45 minutes followed by incubation with Protein A– 10nm Gold Conjugate for 40 minutes at room temperature (Cytodiagnostics, DKU: AC-10-05). Grids were fixed in 1% Glutaraldehyde and 2% PFA in NaCac. The grids were then stained with 4% uranylacetate in 0.15M Oxalic Acid, pH 7, embedded with 2% methylcellulose in 4% uranylacetate, and imaged in a JEOL, JEM1400 is a 120kV Transmission Electron Microscope with a LaB6 emitter and imaged using a Gatan Orius 10.7 megapixel CCD camera. Images were taken at either 25,000X or 40,000X.

### Nano tracking analysis (NTA)

The concentration and size distribution profiles of ExoE-EV were evaluated using a NanoSight NS300 instrument (Malvern, Worcestershire, UK) following manufacturer’s instructions. Videos were recorded at camera level 14. The following post acquisition settings were selected: minimum detection threshold of 3, automatic blur, and automatic minimum expected particle size. Samples were diluted 1:10–1:1000 in PBS and loaded using a syringe pump. For each sample, three 30-second videos were recorded and analyzed in the batch-processing mode using Nano Tracking Analysis (NTA) software.

### Immunoblotting

Whole cell lysates (WCL) and ExoE-EV lysates (EXO) from TSC and SynT cultures were prepared in lysis buffer containing: 25 mM Tris-HCl pH 7.6, 150 mM NaCl, 1% NP-40, 1% sodium deoxycholate, and 0.1% sodium dodecyl sulfate. Both WCL (n = 3) and EXO lysates (n = 3) were sonicated and protein concentration was determined by DC protein assay (Bio-Rad 5000112). 20 μg of each lysate were loaded into a 10% Bio-Rad Criterion gel for sodium dodecyl sulfate-polyacrylamide gel electrophoresis. Protein was transferred to polyvinylidene difluoride membrane (0.2 μm, Bio-Rad 1620177) and blocked in 5% blotting-grade milk (Bio-Rad 1706404) in Tris-buffered saline containing 0.05% Tween 20 (TBS-T). Membranes were incubated with primary anti-CD63 antibody (1:1000, System Biosciences) or Tsg101 (1:250, Santa Cruz Biotechnology, Inc.) in blocking buffer overnight at 4°C. Membranes were washed 3 times in TBS-T and probed with species appropriate secondary antibody (1:100,000, Sigma). Following a final wash in TBS, immunoreactivity was visualized using Western-Lightning chemiluminescent substrate (Perkin-Elmer NEL103E001) per manufacturer’s instructions and exposed to Ultra Blue X-Ray film (Light Labs X-3001).

Sera and ExoE-EVs from pregnant and non-pregnant mice were prepared in 20uL M-PER Mammalian Proteins Extraction Reagent (Thermo Scientific # 78503). Samples were incubated at room temperature for 5 minutes to allow for complete lysis. The lysate was centrifuged at 14,000x g for 10 minutes to remove cell debris. The protein concentration was determined using NanoDrop A280 (NanoDrop 2000, Thermo Scientific). Thirty micrograms of sera and exosome sera lysates were loaded onto a NuPAGE 10% Bis-Tris Protein Gel, 1.5 mm, 10-well (Invitrogen, NP0315BOX). The gel ran for 1 hour at 200V with 1XNuPAGE MOPS SDS running buffer (Invitrogen, NP0001). The proteins from the gel were then transferred onto a polyvinylidene difluoride membrane (Bio-Rad, 162–0177) using chilled 1X NuPage Transfer buffer (Invitrogen, NP0006-1). The membrane was then blocked with 5% blotting-grade nonfat dry milk (Bio-Rad, 1706404EDU) in Tris-buffered saline containing 0.05% Tween-20 (TBS-T) at room temperature for 1 hour with shaking. The membranes were incubated with either primary antibody CD63 (1:250, System Biosciences EXOAB-CD63A-1) Tsg101 (C-2) (1:250, Santa Cruz Biotechnology, Inc. sc-7964) or GRP94 (1:200, Santa Cruz Biotechnology, Inc.). Membranes were washed 4 times in TBS-T (5 minutes per wash with shaking) then incubated with the appropriate secondary antibody: goat anti-rabbit HRP (1:10,000, System Biosciences EXOAB-CD63-1) or anti-mouse IgG HRP (1:10:000, GE Healthcare, NA931V). Membranes were washed with TBS-T as mentioned above and immunoreactivity was visualized using SuperSignal West Femto (Thermo Scientific, 34095) and imaged in an Odyssey FC imager controlled by Image studio software (LI-COR Biosciences, NE, USA).

### RNA extraction, RNA-Seq library construction and sequencing

RNA was purified from ExoE-EV using SeraMir RNA kits (System Biosciences) as previously detailed [7, 14]. RNA-Seq libraries were constructed using modified Illumina adapter methods using the XMIR exosome RNA-Seq Sample Preparation Kit (System Biosciences) and indexed with separate bar codes for multiplex sequencing on an Illumina MiSeq v3 instrument using a 2 x 75 bp paired end run setting. System Biosciences performed all steps of NGS preparation and sequencing.

### Next generation RNA sequence data analyses

The ExoE-EV RNA-Seq data analysis was performed by Maverix Biomics custom exosome RNA-Seq analysis pipeline. Data quality check of the input sequence was first performed using FastQC, an open-source quality control (QC) tool for high-throughput sequence data [15]. Bowtie2 was then used to map the spike-in DNA before the trimming and filtering steps where RNA-Seq reads were preprocessed to improve the quality of data input for read mapping [16]. The open-source tools used for trimming of adapters included FastqMcf, part of the ea-utils package [17], and cutadapt [18], with PRINSEQ [19] used in the quality filtering step. Once the data preprocessing step detected and removed N’s at the ends of reads, trimmed sequencing adapters, and filtered reads for quality and length, FastQC was then re-run to analyze the trimmed reads, allowing a before and after comparison. The sequence reads were merged using SeqPrep [20] then mapped to the reference genome using Bowtie [21], followed by generation of a mapping rate summary. Using the open-source software BEDTools [22] and SAMtools [23], read alignment and read coverage tracks were generated and deployed to the customized UCSC genome browser [24]. Quantitation and alignment to miRbase [25], a built-in UCSC genome browser tool, increases the likelihood that only reads aligned to mature miRNAs, rather than transcript precursors or non-miRNA loci, were counted during abundance determination [26]. The final steps of the analysis were abundance determination and Abundance levels for ncRNAs (miRNAs, tRNAs, rRNAs, lincRNAs, piRNAs, and snoRNAs), antisense transcripts, coding genes and repeat elements (LTR, LINE, SINE, and tandem repeats) were determined, then a summary of reads overlapping each of these annotations in the reference genome was created using SAMtools and provided for visualization in pie charts using R, a software environment for statistical computing and graphics [27] Finally, differences in expression of ncRNA were calculated and differential expression analysis was performed using DEseq [28]; adjusted P-values were calculated using Benjamini-Hochberg procedure [28]. The authors deposited the data in the NCBI’s Gene Expression Omnibus database. (*Accession number to be provided once manuscript accepted*.)

### Quantitative RT-PCR analysis

RNA was purified from the ExoE-EV using SeraMir RNA kits (System Biosciences), as described above from independent cultures of TSC (n = 5) and SynT (n = 5) cells. Briefly, 100 ng of purified RNA from each sample was poly-adenylated, and cDNA was synthesized according to the QuantimiR protocol (System Biosciences). NP (n = 8) and GD 14.5 (n = 8) RNA was isolated as described above. Purified RNA from each serum sample was reverse-transcribed into cDNA. Relative miRNA abundance was determined with Maxima SYBR Green detection (Fermentas, Inc.) using Universal reverse primer and a forward primer designed for miRNAs of interest (S1 Table) and an HT 7900 sequence detection system (Applied Biosystems). Samples were run in triplicate. Mmu-miR-744-5p expression was used as an internal reference as its expression did not change across groups (TSC/SynT and NP/GD 14.5) as determined by both RNA-Seq and qRT-PCR. The relative miRNA abundance was calculated with the delta-delta CT method [29, 30]. Statistical analysis and graphs of qRT-PCR data was performed using GraphPad Prism 5. Nonparametric Mann-Whitney U tests were performed between groups (TSC v. SynT; NP v. GD 14.5) with a significance level set at p<0.05.

### MicroRNA target prediction and functional annotation using multiMiR and KEGG

Predicted miRNA-target interactions were determined by utilizing the multiMiR R package to simultaneously query eight publicly available databases: DIANA-microT-CDS, EIMMo, MicroCosm, miRanda, miRDB, PicTar, PITA, and TargetScan [31, 32]. MultiMiR queries were performed on April 20, 2015 and run with the default settings, with the modification of ‘predicted’ targets instead of the program default ‘validated’. The resultant data was filtered using R by first selecting all unique Ensembl IDs returned as predicted targets of selected miRNAs of interest. For each miRNA, the number of databases (0–8) predicting an interaction with a given target was counted. Targets with three or greater of the eight databases predicting the interaction were assigned the value “1” while those with two or fewer were assigned the value “0”. All predicted targets with at least one miRNA with a highly-predicted miRNA-target interaction (predicted by three or more databases) were imported into the Functional Annotation feature of the DAVID Bioinformatics Resource 6.7 to query Kyoto Encyclopedia of Genes and Genomes (KEGG) pathways [33–35]. The list of predicted targets was uploaded as a gene list with Ensembl gene ID as the identifier and mus musculus selected as both the species and background. No additional parameters were provided.

## Results

### Characterization of trophoblast-derived and pregnancy-associated ExoE-EVs

**Fig 1:**
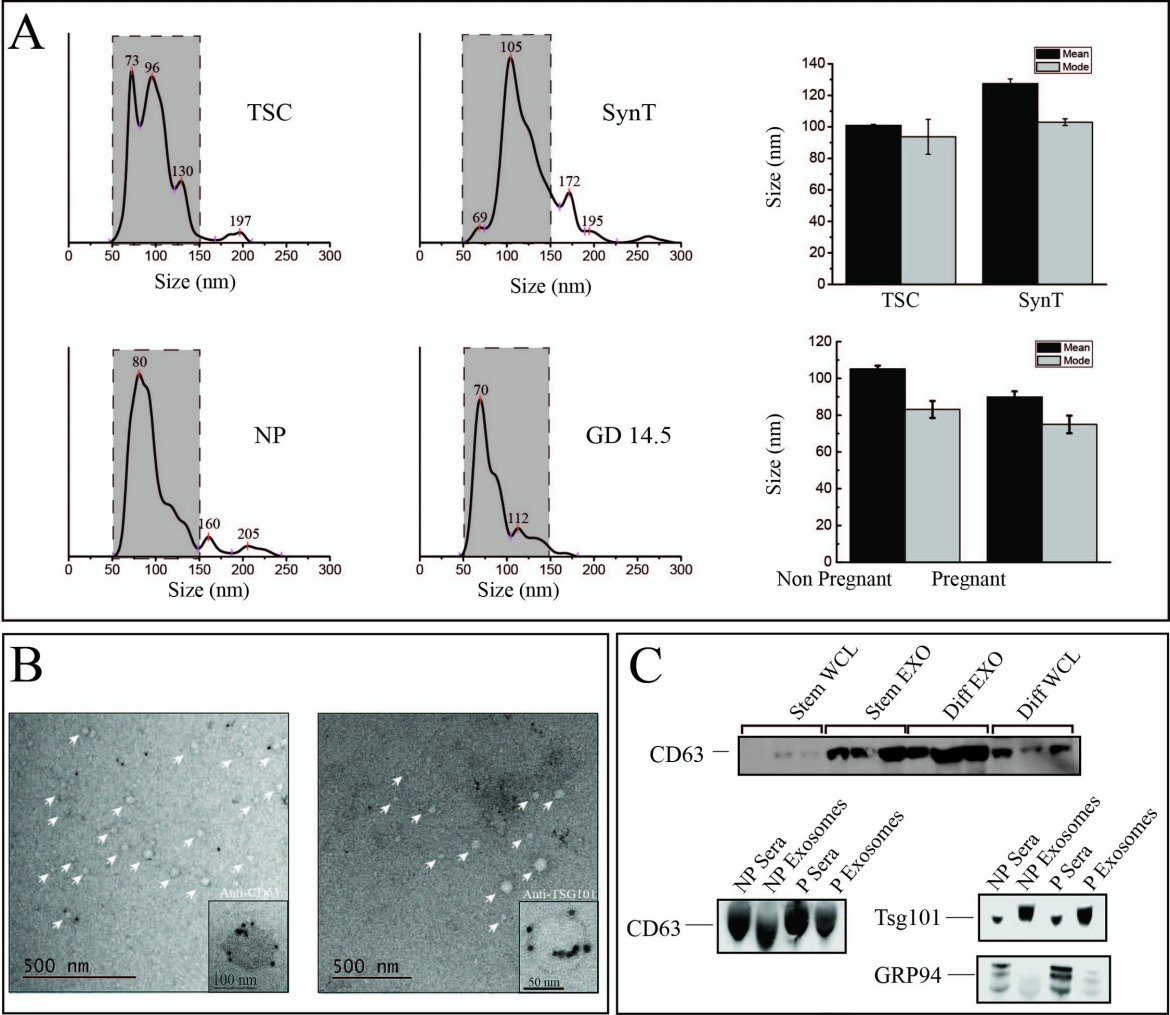
Characterization of ExoE-EVs. (A) NTA analysis of ExoQuick isolated exosome enriched extracellular vesicles (ExoE-EVs) from conditioned media of trophoblast stem cells (TSC) and syncytial cells (SynT) and murine sera of non-pregnant (NP) and pregnant mice at gestational day 14.5 (GD 14.5). Dashed gray boxes represent enrichment of exosomes in the 50-150nm range; TSC (94.9%), SynT (80.5%), NP (92.8%) and GD 14.5 (81.3%). The mean and mode of each population is also provided. (B) Representative immunoelectron micrographs of serum ExoE-EV for the exosome markers CD63 and TSG101. (C) Representative immunoblot of whole cell (WCL), exosomal (ExoE-EV) protein lysates from TSC and SynT *in vitro* cultures (n = 3, group) and from NP and P (GD14.5) sera and ExoE-EV.

To characterize the EVs isolated using ExoQuick, samples were examined by nano tracking analysis (NTA), electron microscopy (EM), and immunoblot analysis (Fig 1). ExoQuick was chosen over ultracentrifugation and gradient methods given the limited starting material of sera that can be collected from pregnant mice. The majority of isolated EVs are between 50–150 nm with median and modes of representative preparations shown in Fig 1A. All four groups had an enrichment of exosomes (50–150 nm) ranging from 80–95% of the EVs isolated [36]. ImmunoEM was performed on ExoE-EVs derived from mouse serum showing morphology and size consistent with previously reported studies [36, 37] (Fig 1B). The exosomal markers, CD63 and Tsg 101, are detected in ExoQuick EV material and compared to whole cell lysate (WCL) or neat sera, respectively (Fig 1C). Based on the criteria of size, morphology, and protein markers, the EV material used for these analyses are composed predominately of exosomes and hence are referred to as ExoE-EVs.

#### RNA sequencing, abundance, and differential abundance

RNA-Seq was performed to generate a preliminary, unbiased description of the total RNA composition (TSC, SynT, NP, and GD 14.5, n = 3 biological replicates per group) of trophoblast and pregnancy ExoE-EVs. This schema allowed for the identification of all RNAs contained within each group, as well as comparison of the differential abundance of RNAs between groups (TSC vs. SynT and NP vs. GD 14.5).

**Fig 2:**
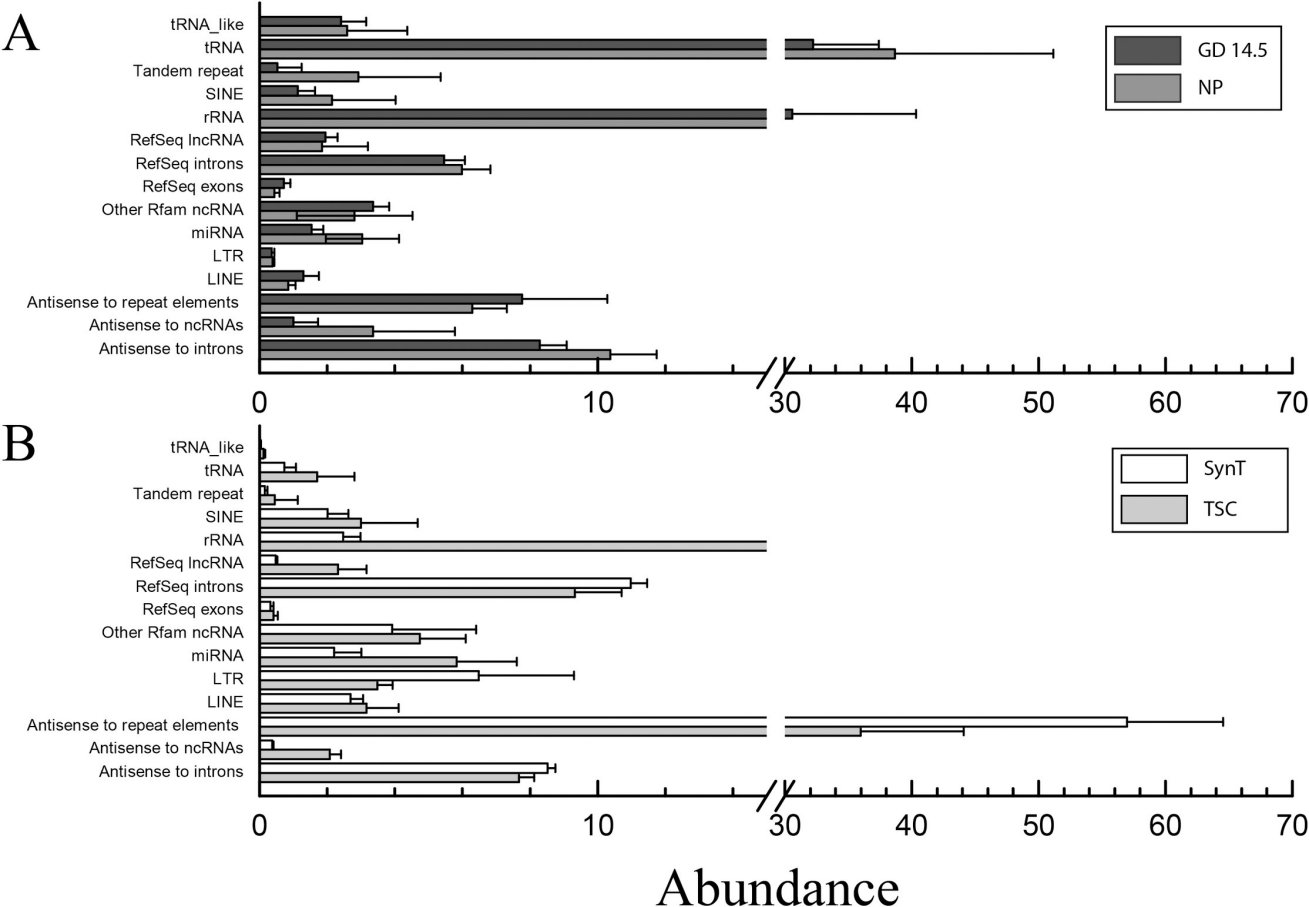
Abundance of RNA by type in murine trophoblast-derived and serum ExoE-EVs. (A) For the ExoE-EVs from the trophoblast cell lines, the prominent RNA species were antisense to repeat elements, rRNA, RefSeq introns, antisense to introns, and miRNA. (B) RNA categories in the murine sera with the highest abundance were tRNA, rRNA, antisense to introns, antisense to repeat elements, RefSeq introns, and miRNA.

Quality, trimming, and read mapping of sequenced RNAs are summarized and presented in S1 Table. The abundance of each type of RNA was determined following mapping. For the ExoE-EVs from the trophoblast cell lines, the prominent RNA species were antisense to repeat elements, rRNA, RefSeq introns, antisense to introns, and miRNAs (Fig 2A). RNA categories with the highest abundance in the murine sera were tRNA, rRNA, antisense to introns, antisense to repeat elements, RefSeq introns and miRNA (Fig 2B). Due to the increasing body of evidence demonstrating gene expression altering and/or immunomodulatory potential of miRNAs on target cells, miRNAs were the focus for the remainder of the analysis in this report. First, the differentially abundant miRNAs between TSC and SynT samples were determined and the miRNAs with the greatest fold change are shown in Table 1. Of these, four miRNAs reached statistical significance with mmu-miR-322-3p and mmu-miR-450a-5p having increased abundance with syncytial differentiation, whereas mmu-miR-5099 and mmu-miR-1198-5p were decreased with TSC to SynT differentiation (p<0.05, n = 5). In addition to those altered with differentiation, we were also interested in those expressed at the highest level in the SynT trophoblast ExoE-EVs as these would likely represent the most abundant placental exosomes released into the maternal circulation. Table 2 lists the most abundant SynT miRNAs. We next looked at the ExoE-EV miRNAs increased in pregnancy. The miRNAs with the largest increased fold change (> 2.15 log_2_ selected for convenience of numbers and a natural breakpoint in the data) in GD 14.5 compared to NP mice are shown in Table 3. However, there were no statistically significant differences between the two groups, likely due to the small sample size. Interestingly, fifteen mature miRNAs were detected only in serum ExoE-EV of GD 14.5 and not in NP mice (Table 4), which would be consistent with these miRNAs being pregnancy-specific and therefore potentially originating from the placenta. Six of these miRNAs are located on chromosome 12 with two pairs of clustered (<10 kb) miRNAs: mmu-mir-431 with mmu-mir-665 and mmu-mir-679 with mmu-mir-667. Finally, we were interested in those miRNAs that were at higher levels in sera from GD 14.5 compared to NP and that were also abundant in the trophoblast-derived ExoE-EVs. Those that met this criterion with the highest abundance were mmu-miR-322-3p, mmu-miR-292-5p,3p and mmu-miR-183-5p.

**Table 1:** Differentially abundant mature microRNAs in exosomes from TSC and SynT trophoblasts.

miRNA ID	Chrom	TSC	SynT	Fold change	Adj. p-value
**INCREASED ABUNDANCE**					
**mmu-miR-6538**	Chr 12	0.23	10.91	5.58	0.50191
**mmu-miR-503-5p**	Chr X	70.10	1735.62	4.63	0.44395
**mmu-miR-322-3p**	Chr X	56.26	1364.55	4.60	**0.00013**
**mmu-miR-450a-5p**	Chr X	5.05	103.66	4.36	**0.00624**
**mmu-miR-5114**	Chr 19	0.26	2.99	3.54	1.00000
**mmu-miR-1981-5p**	Chr 1	19.05	219.85	3.53	0.13604
**mmu-miR-7073-5p**	Chr 8	0.11	0.90	3.04	1.00000
**mmu-miR-542-3p**	Chr X	36.09	291.37	3.01	1.00000
**mmu-miR-351-3p**	Chr X	5.75	45.82	3.00	1.00000
**mmu-miR-542-5p**	Chr X	0.11	0.79	2.86	1.00000
**mmu-miR-7226-3p**	Chr 4	0.23	1.65	2.86	1.00000
**mmu-miR-450b-5p**	Chr X	24.66	177.10	2.84	0.44395
**DECREASED ABUNDANCE**					
**mmu-miR-335-3p**	Chr 6	39.94	0.40	-6.64	0.44395
**mmu-miR-335-5p**	Chr 6	18.89	0.32	-5.87	0.06951
**mmu-miR-34b-5p**	Chr 9	47.12	0.88	-5.74	0.70626
**mmu-miR-294-3p**	Chr 7	3037.59	73.94	-5.36	1.00000
**mmu-miR-5099**	Chr 12	64.22	1.71	-5.23	**0.00013**
**mmu-miR-291a-3p**	Chr 7	985.25	28.14	-5.13	1.00000
**mmu-miR-1198-5p**	Chr X	34.03	1.08	-4.98	**0.00071**
**mmu-miR-218-5p**	Chr 5/11	3.20	0.11	-4.92	1.00000
**mmu-miR-292-3p**	Chr 7	4186.87	161.43	-4.70	1.00000
**mmu-miR-532-3p**	Chr X	4.33	0.17	-4.65	1.00000
**mmu-miR-6239**	Chr 14	86.71	3.63	-4.58	1.00000
**mmu-miR-674-5p**	Chr 2	4.52	0.19	-4.56	1.00000

Fold change = log_2_ fold change

**Table 2:** Most abundant mature microRNAs in ExoE-EVs derived from SynT differentiated TSCs.

miRNA ID	Chromosome	TSC	SynT	Fold Change
**mmu-miR-122-5p**	Chr 18	39229.22	12868.24	-1.61
**mmu-miR-378a-3p**	Chr 18	2201.70	11116.78	2.34
**mmu-miR-30d-5p**	Chr 15	2970.53	6685.98	1.17
**mmu-miR-292-5p**	Chr 7	42021.98	4547.92	-3.21
**mmu-miR-148a-3p**	Chr 6	6982.50	3796.35	-0.88
**mmu-miR-183-5p**	Chr 6	2306.61	2361.57	0.03
**mmu-miR-320-3p**	Chr 14	1076.28	2232.53	1.05
**mmu-miR-92a-3p**	Chr 14	7163.29	1922.49	-1.90
**mmu-miR-532-5p**	Chr X	7530.28	1891.93	-1.99
**mmu-miR-503-5p**	Chr X	70.10	1735.62	4.63
**mmu-miR-30a-5p**	Chr 1	1852.85	1590.52	-0.22
**mmu-miR-378d**	Chr 10	380.88	1510.57	1.99
**mmu-miR-322-3p**	Chr X	56.26	1364.55	4.60
**mmu-miR-298-5p**	Chr 2	345.95	1253.61	1.86
**mmu-miR-22-3p**	Chr 11	1113.76	1246.69	0.16
**mmu-miR-151-3p**	Chr 15	1530.87	746.28	-1.04
**mmu-miR-378c**	Chr 14	175.86	705.17	2.00
**mmu-miR-669c-5p**	Chr 2	1222.48	703.74	-0.80
**mmu-miR-7a-5p**	Chr 7 or 13	3127.45	555.56	-2.49
**mmu-miR-92a-3p**	Chr X	2104.96	520.11	-2.02
**mmu-miR-101b-3p**	Chr 19	511.10	516.93	0.02
**mmu-miR-101a-3p**	Chr 4	289.53	480.29	0.73
**mmu-miR-24-3p**	Chr 8 or 13	207.62	476.85	1.20
**mmu-miR-293-5p**	Chr 7	3163.89	453.16	-2.80
**mmu-miR-501-3p**	Chr X	744.34	434.02	-0.78

Fold change = log_2_ fold change

**Table 3:** Mature microRNAs with greatest positive fold change in ExoE-EVs from non-pregnant to pregnant mice.

miRNA ID	Chromosome	NP	Day14.5	Fold change	Adj. p-value
**mmu-miR-147-3p**	Chr 2	0.69	24.43	5.15	1.00000
**mmu-miR-450b-5p**	Chr X	1.24	40.60	5.04	0.44395
**mmu-miR-323-3p**	Chr 12	0.34	9.04	4.72	1.00000
**mmu-miR-543-3p**	Chr 12	2.13	54.67	4.68	1.00000
**mmu-miR-149-5p**	Chr 1	1.03	20.39	4.31	1.00000
**mmu-miR-542-3p**	Chr X	2.13	38.10	4.16	1.00000
**mmu-miR-182-5p**	Chr 6	8.06	127.20	3.98	1.00000
**mmu-miR-495-3p**	Chr 12	0.69	9.65	3.81	1.00000
**mmu-miR-293-5p**	Chr 7	0.89	11.40	3.67	1.00000
**mmu-miR-183-5p**	Chr 6	22.91	279.33	3.61	1.00000
**mmu-miR-341-3p**	Chr 12	0.69	8.21	3.58	NA
**mmu-miR-433-3p**	Chr 12	5.45	57.85	3.41	1.00000
**mmu-miR-409-3p**	Chr 12	3.92	35.04	3.16	1.00000
**mmu-miR-219a-1-3p**	Chr 17	0.89	7.08	2.98	1.00000
**mmu-miR-335-3p**	Chr 6	0.34	2.36	2.78	0.44395
**mmu-miR-351-5p**	Chr X	5.31	33.52	2.66	1.00000
**mmu-miR-195a-3p**	Chr 11	2.27	13.76	2.60	1.00000
**mmu-miR-208a-3p**	Chr 14	0.69	3.79	2.46	NA
**mmu-miR-291b-5p**	Chr 7	1.45	7.92	2.45	1.00000
**mmu-miR-212-5p**	Chr 11	0.34	1.85	2.43	1.00000
**mmu-miR-351-3p**	Chr X	0.89	4.63	2.37	1.00000
**mmu-miR-615-3p**	Chr 15	1.24	6.38	2.37	1.00000
**mmu-miR-322-3p**	Chr X	25.95	127.92	2.30	**0.00013**
**mmu-miR-134-5p**	Chr 12	18.55	84.73	2.19	1.00000
**mmu-miR-292-5p**	Chr 7	37.82	171.18	2.18	1.00000

Fold change = log_2_ fold change

**Table 4:** Mature microRNAs unique to serum ExoE-EVs of pregnant mice.

miRNA ID	Chromosome	NP	GD14.5
**mmu-miR-431-3p**	Chr 12	0	19.41
**mmu-miR-292-3p**	Chr 7	0	7.18
**mmu-miR-679-5p**	Chr 12	0	6.99
**mmu-miR-370-3p**	Chr 12	0	6.38
**mmu-miR-485-3p**	Chr 12	0	5.95
**mmu-miR-664-3p**	Chr 1	0	5.73
**mmu-miR-483-3p**	Chr 7	0	4.63
**mmu-miR-877-3p**	Chr 17	0	4.33
**mmu-miR-467e-5p**	Chr 2	0	3.39
**mmu-miR-152-5p**	Chr 11	0	3.29
**mmu-miR-6240**	Chr 5	0	3.28
**mmu-miR-665-3p**	Chr 12	0	3.28
**mmu-miR-667-3p**	Chr 12	0	3.28
**mmu-miR-143-5p**	Chr 18	0	2.98
**mmu-miR-3084-5p**	Chr 19	0	2.86

Fold change = log_2_ fold change

#### Abundance of microRNAs mmu-miR-322-3p and mmu-miR-322-5p increases with syncytial differentiation *in vitro* and with pregnancy *in vivo*

**Fig 3:**
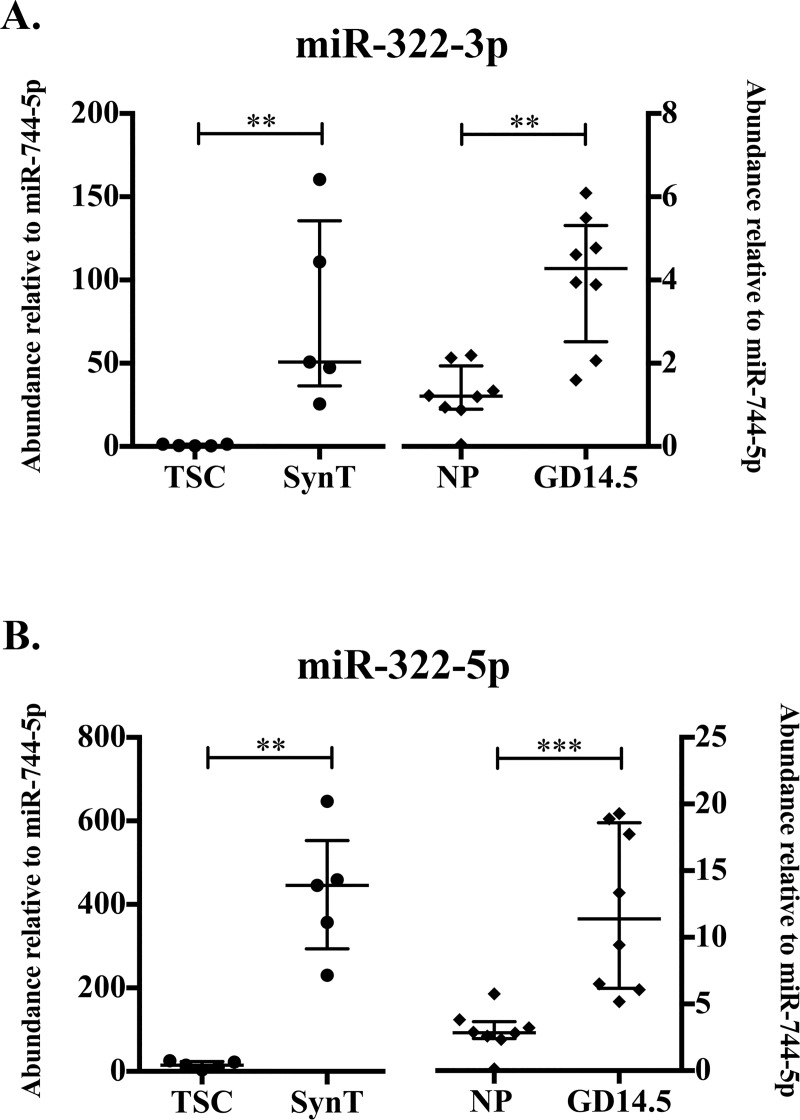
Abundances of microRNAs mmu-miR-322-3p and mmu-miR-322-5p increase with syncytial differentiation *in vitro* and with pregnancy *in vivo*. (A) Abundance of mmu-miR-322-3p relative to mmu-miR-744-5p determined by qRT-PCR in TSC and SynT (right, solid diamond, n = 5, p = 0.0079) and NP and GD14.5 (left, solid circle, n = 8, p = 0.0019). (B) Abundance of mmu-miR-322-5p relative to mmu-miR-744-5p determined by qRT-PCR in TSC and SynT (right, n = 5, p = 0.0079) and NP and GD14.5 (left, n = 8, p = 0.0003). Error bars represent median ± interquartile range (** p<0.01; *** p<0.001).

RNA-Seq of *in vitro-* and *in vivo-*derived ExoE-EVs revealed a subset of miRNAs that are likely to be associated with trophoblasts, pregnancy, or both. RNA sequencing presented in this study identified mmu-miR-322-3p as significantly more abundant in SynT compared to TSC ExoE-EVs (Table 1). These results subsequently led us to focus on both of the mature miRNAs from the mmu-mir-322 precursor. Abundance of mmu-miR-322-3p and mmu-miR-322-5p exhibited a trend of increasing with pregnancy (log_2_ fold change = 2.30 and 1.24, respectively), and mmu-miR-322-5p exhibited a trend in increased abundance with syncytial differentiation (log_2_ fold change = 2.17). To further quantify and validate the abundance of mature mmu-mir-322 products in our trophoblast- and pregnancy-derived samples, qRT-PCR was performed on a larger sample size of independent samples. Using this method, the relative abundance of mmu-miR-322-3p increased in SynT differentiation (p = 0.0079, n = 5) *in vitro* cell culture and *in vivo* during pregnancy (p = 0.0019, n = 8) (Fig 3A). Significant differences in the relative abundance of mmu-miR-322-5p were detected with both syncytial differentiation (p = 0.0079) and pregnancy (p = 0.0003) by qRT-PCR (Fig 3B). These results suggest that subsets of ExoE-EV miRNAs (mmu-mir-322 products) are increased in both syncytial differentiation and pregnancy.

#### X-chromosome miRNA cluster members increase in abundance with syncytial differentiation and pregnancy

**Fig 4:**
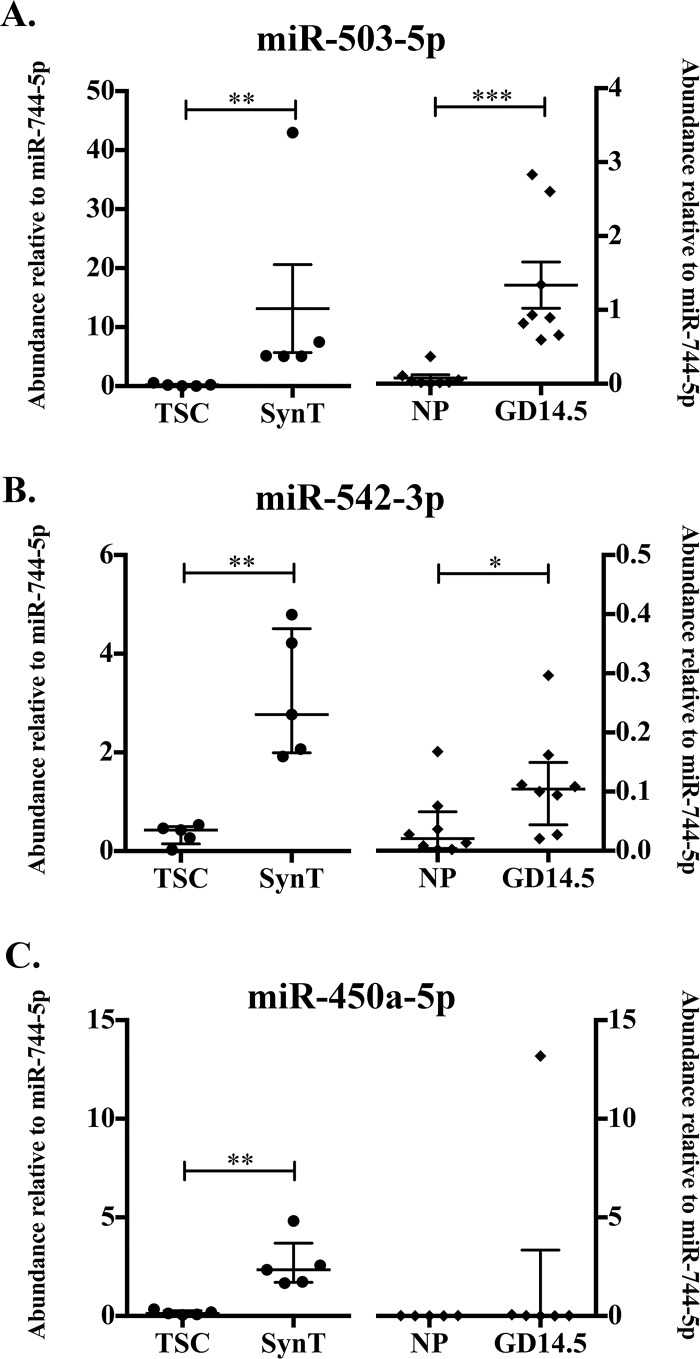
Abundance of X-Chromosome microRNA cluster members in Exo-EVs increases with synctial differentiation in pregnancy. (A) Abundance of mmu-miR-503-5p relative to mmu-miR-744-5p determined by qRT-PCR in TSC and SynT (right, solid diamond, n = 5, p = 0.0079) and NP and GD14.5 (left, solid circle, n = 8, p = 0.0002). (B) Abundance of mmu-miR-542-3p relative to mmu-miR-744-5p determined by qRT-PCR in TSC and SynT (right, n = 5, p = 0.0079) and NP and GD14.5 (left, n = 8, p = 0.0499). (C) Abundance of mmu-miR-450a-5p relative to mmu-miR-744-5p determined by qRT-PCR in TSC and SynT (right, n = 5, p = 0.0079) and NP and GD14.5 (left, n = 8). Error bars represent median ± interquartile range. (* p< 0.05; ** p<0.01; *** p<0.001).

In addition to the X-chromosome derived mmu-miR-322-3p and mmu-miR-322-5p, abundance of mir-322/424 cluster member, mmu-miR-450a-5p, was found to be significantly increased in SynT compared to TSC and exhibited an increase in pregnancy samples (log_2_ fold change = 2.03; Table 3). X-chromosome miRNAs mmu-miR-503-5p, mmu-miR-542-3p, and mmu-miR-450a-5p also exhibited non-significant increases in abundance in GD 14.5 compared to NP. These additional X-chromosome miRNAs were selected for qRT-PCR analysis to determine relative abundance using a larger sample size. This analysis demonstrated a significant increase in miRNA abundance in SynT compared to TSC for all three-cluster members: mmu-miR-503-5p, mmu-miR-542-3p, and mmu-miR-450-5p (p = 0.0079, n = 5; Fig 4). The relative abundances of mmu-miR-503-5p and mmu-miR-542-3p were increased in pregnant compared to NP ExoE-EVs (Fig 4). These data suggest that abundances of mmu-miR-322-3p, mmu-miR-322-5p and closely clustered X-chromosome miRNAs are associated with trophoblast syncytial differentiation and pregnancy.

#### MicroRNAs mmu-miR-30d-5p and mmu-miR-5099 are differentially abundant in syncytialized compared to stem trophoblast ExoE-EVs

**Fig 5:**
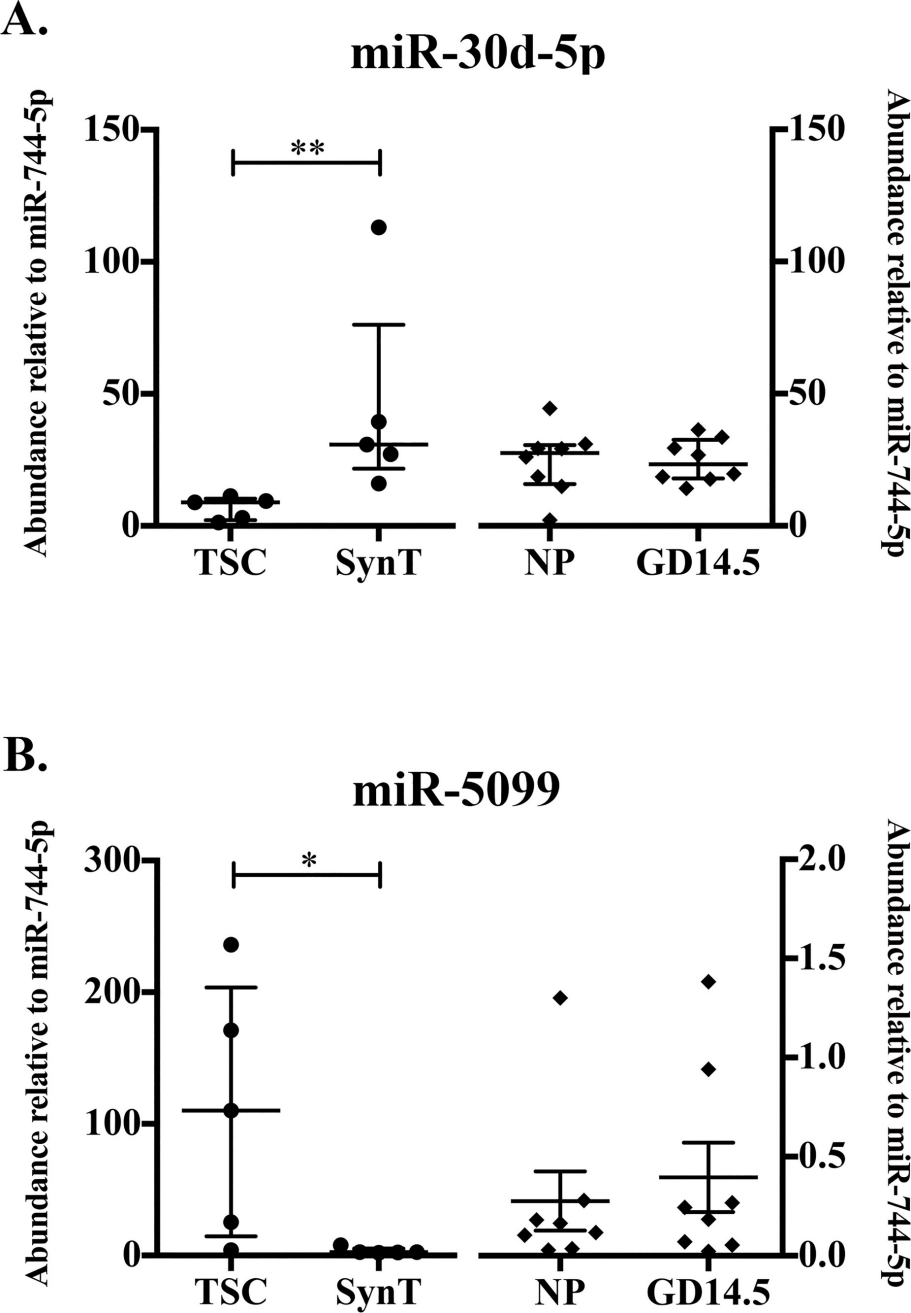
MicroRNAs Mmu-miR-30d-5p and Mmu-miR-5099 are differentially abundant in syncytialized compared to stem trophoblast ExoE-EVs. (A) Abundance of mmu-miR-30d-5p relative to mmu-miR-744-5p determined by qRT-PCR in TSC and SynT (right, n = 5, p = 0.0079) and NP and GD14.5 (left, n = 8). (B) Abundance of mmu-miR-5099 relative to mmu-miR-744-5p determined by qRT-PCR in TSC and SynT (right, n = 5, p = 0.0159) and NP and GD14.5 (left, n = 8). Error bars represent median ± interquartile range. (* p< 0.05; ** p<0.01; *** p<0.001).

Additional miRNAs, mmu-miR-30d-5p and mmu-miR-5099, were selected for further investigation by qRT-PCR. Mmu-miR-30d-5p was selected since it was the third most abundant miRNA in SynT exosomes as determined by RNA-Seq (Table 2). Using qRT-PCR, we found that the relative abundance of mmu-miR-30d-5p increased significantly with syncytial differentiation (p = 0.0079, n = 5; Fig 5A). No differences were detected in the relative abundance of mmu-miR-30d-5p between pregnant and NP mice (Fig 5A). Our RNA-Seq analysis revealed that mmu-miR-5099 decreased significantly with syncytial differentiation. Although downregulation of miRNAs during syncytial differentiation was not a focus of this study, it was validated to ensure we could detect changes in both directions. As expected, mmu-miR-5099 was significantly downregulated in SynT compared to TSC (p = 0.0159, n = 5; Fig 5B). No differences were observed in the relative abundance of mmu-miR-5099 between pregnant and NP mice (Fig 5B). These data demonstrate that the differentiation status of cultured trophoblasts can result in significant quantitative changes in abundances of individual miRNAs.

#### Embryonic stem cell/fetal-associated microRNAs increase during pregnancy in circulating ExoE-EVs

**Fig 6:**
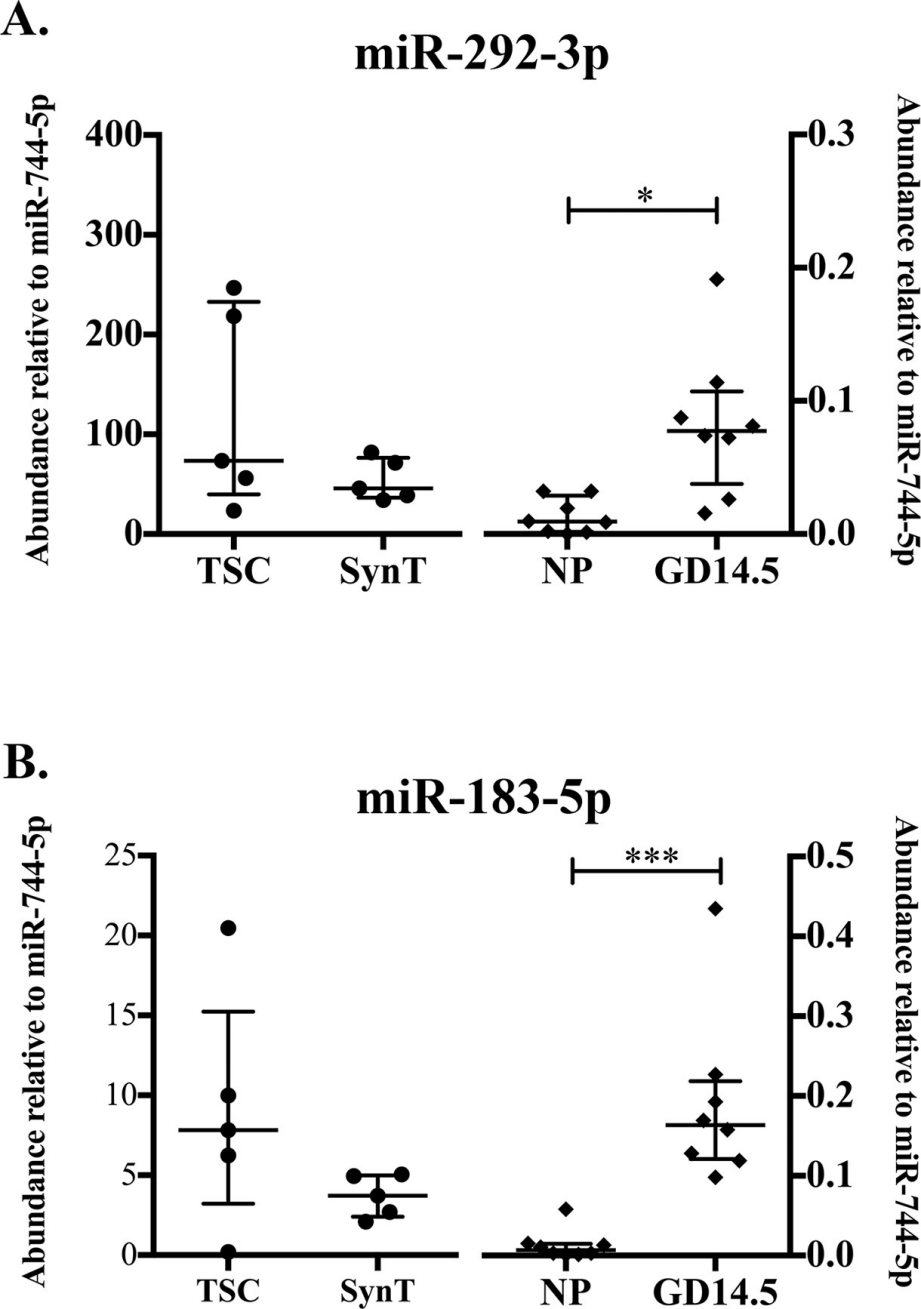
Embryonic stem cell/fetal-associated MiRNAs increase during pregnancy in circulating ExoE-EVs. (A) Abundance of mmu-miR-292-3p relative to mmu-miR-744-5p determined by qRT-PCR in TSC and SynT (right, solid diamonds, n = 5) and NP and GD14.5 (left, solid circles, n = 8, p = 0.0030). (B) Abundance of mmu-miR-183-5p relative to mmu-miR-744-5p determined by qRT-PCR in TSC and SynT (right, n = 5) and NP and GD14.5 (left, n = 8, p = 0.0002). Error bars represent median ± interquartile range. (* p< 0.05; ** p<0.01; *** p<0.001).

We next sought to confirm the regulation of the miRNAs that are higher in pregnancy and expressed highly in SynT trophoblast ExoE-EVs, specifically mmu-miR-292-3p and mmu-miR-183-5p. Of this cluster, mmu-miR-292-3p was of particular interest because it was detected by RNA-Seq in pregnant mice and not in NP mice (Table 4). qRT-PCR analysis of mmu-miR-292-3p revealed no significant differences in relative abundance between TSC and SynT. However, a significant increase in relative abundance was detected between GD 14.5 and NP (p = 0.003, n = 8; Fig 6A). For mmu-miR-183-5p, a significant difference in abundance was also observed between GD 14.5 and NP (p = 0.0002, n = 8; Fig 6B). Here we demonstrate increased abundance of ESC/embryo-associated miRNAs, mmu-miR-292-3p and mmu-miR-183-5p, in the circulation of GD 14.5 compared to NP mice. Whether these are coming from the fetus itself, the placenta, or maternal stem cells is still to be determined.

#### MicroRNA-target interaction prediction using multiMiR

Selected X-chromosome miRNAs including mmu-miR-322-3p, mmu-miR-322-5p, mmu-miR-503-5p, and mmu-miR-542-3p were queried, using the multiMiR package, to predict their potential mRNA targets. These miRNAs were selected because they belong to the same miRNA cluster, are increased both with SynT differentiation and in pregnancy, are known to be expressed in mouse placenta, and their human orthologs are expressed in human placenta [7, 38].

The multiMiR package queries eight independent databases (DIANA-microT-CDS, EIMMo, MicroCosm, miRanda, miRDB, PicTar, PITA, and TargetScan) for predicted targets. For each miRNA, hundreds to thousands of unique mRNA targets were identified by the eight databases. In order to reduce this to a meaningful list with more confidence in the miRNA-target prediction, targets with three or more of the eight databases predicting the miRNA-target interaction were considered to be of higher confidence. The high-confidence miRNA-target interactions are presented in the supplemental material (S2 Table). In total, 855 unique targets were identified.

#### MultiMiR predicted targets reveal significant targeting of intracellular signaling and ubiquitin-mediated proteolysis pathways by X-chromosome microRNAs

All unique targets with at least one high-confidence interaction predicted for mmu-miR-322-3p, mmu-miR-322-5p, mmu-miR-503-5p, or mmu-miR-542-3p from MultiMiR analysis were imported into the DAVID Bioinformatics Resource 6.7 for KEGG pathway analysis. Of the 855 unique Ensembl IDs imported, 846 matched DAVID IDs and were included in the subsequent KEGG analysis. All KEGG pathways significantly targeted (p<0.05) by predicted miRNA-target interactions are summarized in S3 Table. The pathway most targeted by the X chromosome cluster miRNAs is identified as Pathways in Cancer (S2 Fig). Interestingly, of the additional significantly targeted KEGG pathways, many are intracellular signaling pathways including MAPK Signaling (S3 Fig) and Focal Adhesion Pathways (S4 Fig). KEGG Pathway analysis also reveals that multiple transcripts involved in ubiquitin-mediated proteolysis were predicted targets of miRNAs mmu-miR-322-3p, mmu-miR-322-5p, mmu-miR-503-5p, and/or mmu-miR-542-3p (S5 Fig) including multiple members of the cullin family that were redundantly targeted by X-Chromosome miRNAs. Table 5 shows the number of MultiMiR databases that predicted an interaction of each miRNA. Each targeting and inhibition of critical signaling molecules may have a multitude of effects on cellular function depending on the targeted cell receiving the miRNA’s cullin family member.

**Table 5:** Murine trophoblast exosome-associated microRNAs target cullins.

	mmu-miR-322-3p	mmu-miR-322-5p	mmu-miR-503-5p	mmu-miR-542-3p
Cul 2	1	7	1	-
Cul3	1	-	-	-
Cul4a	2	1	3	1
Cul4b	3	1	-	-
Cul5	2	1	-	-

These results highlight important signaling pathways and protein family members likely to be targeted by X-chromosome cluster miRNAs upon target cell acquisition of circulating trophoblast-derived exosomes.

## Discussion

Placenta-derived EVs, specifically exosomes, have been implicated in maternal-fetal communication where interactions occur with maternal cells including decidual cells, circulating immune cells, and endothelial cells [3, 39, 40]. Currently, murine models are often used to study pregnancy and maternal-fetal tolerance, due to the challenges of carrying out definitive studies in humans. In an effort to understand the role of placental exosomes in pregnancy, a comprehensive study of the total RNA and miRNA cargo of trophoblast-derived and pregnancy-associated exosome enriched EVs was performed in a murine model. Identification of RNA and miRNA cargo that can mediate cell-cell communication between the placenta/fetus and the mother will advance our ability to examine the role of exosomes in pregnancy and pregnancy-related diseases.

We identified five mature miRNAs (mmu-miR-322-3p, mmu-miR-322-5p, mmu-miR-503-5p, mmu-miR-542-3p, and mmu-miR-450-5p) from the mir-mmu-322 X chromosome cluster that were significantly increased in ExoE-EVs with trophoblast syncytial differentiation, and fifteen miRNAs that were unique to pregnancy ExoE-EVs compared to NP controls. To assess the potential targets of these miRNAs, we utilized the bioinformatics tool MultiMiR to simultaneously query eight publicly available miRNA target databases. X-chromosome miRNAs mmu-miR-322-3p, mmu-miR-322-5p, mmu-miR-503-5p, and mmu-miR-542-3p were predicted to target intracellular signaling pathways (MAPK and focal adhesion) and ubiquitin-mediated proteolysis, and specifically several cullin family proteins. Pathways such as MAPK signaling and focal adhesion are critical to the functions of many cells, including trophoblasts, endothelial cells and immune cells, all potential cellular targets of circulating exosomes [41, 42]. Trophoblast-derived and pregnancy-associated exosomes therefore may exert key regulatory effects on the intracellular signaling of these as well as other target cells and impact the maintenaince and ultimate success of pregnancy.

Exosome-mediated targeting of the ubiquitin-mediated proteolysis pathway may alter protein localization and/or stability in target cells. Cullins carry crucial roles in the post-translational modification of proteins involving ubiquitin [43]. We identified that several different cullins are predicted targets of the mmu-mir-322 family members (mmu-miR-322-3p, mmu-miR-322-5p, mmu-miR-503-5p, and mmu-miR-542-3p), with each predicted to target at least one cullin family member (Table 5). The targeting of cullin-2 (Cul2) by mmu-miR-322-5p was especially highly predicted with seven out of eight databases forecasting the interaction. The interaction between the human ortholog of mmu-miR-322-5p, hsa-miR-424-5p, and Cul2 has been validated by multiple methods [44]. It has also been demonstrated that Cul2 is a critical mediator of HIF-1α degradation in normoxia [44]. Thus, targeting and repression of Cul2 translation by mmu-miR-322-5p may impact cell function by allowing for increased levels of HIF-1α. Given that HIFs are known critical mediators of trophoblast differentiation and function [45], it is possible that trophoblastic exosomes expressing hsa-miR-424/mmu-miR-322 can alter gene expression and cellular functions in multiple cell types, including endothelial, immune, and trophoblast cells.

In T cells, independent of hypoxia, Cul2 and Cul3 regulate interleukin-2 production (IL-2). Specifically, loss of Cul2 or Cul3 resulted in an increase in IL-2 production upon T cell receptor stimulation suggesting that repression of these cullins would impact intracellular signaling cascades to allow for increased cytokine release [46, 47]. Pregnancy is known to be a state of enhanced levels of cytokines [48]. Because all of these X-chromosome cluster miRNAs may be present simultaneously, exosomes delivered to recipient cells could repress translation of multiple cullins. Given the potential cellular effects of cullins and HIFs on a wide variety of cell types, further investigation is necessary to determine the net biological effect of these miRNAs on the predicted targets.

As expected we identified the expression of the placental miRNA mmu-miR-322-5p in our trophoblast-derived ExoE-EVs. The human ortholog of mmu-miR-322-5p, hsa-miR-424-5p, is known to be expressed in human trophoblasts, regulated by hypoxia, increased with *in vitro* syncytialization, and targets fibroblast growth factor receptor 1 (FGFR1) [49, 50]. This is consistent with our findings that mmu-miR-322-5p increases with syncytialization in a murine *in vitro* model. Together with previous data, these findings are consistent with the proposed role for hsa-miR-424/mmu-miR-322 in regulating trophoblast differentiation toward a syncytial fate [51]. In addition, four X cluster members (mmu-miR-322-3p, mmu-miR-322-5p, mmu-miR-503-5p, and mmu-miR-542-3p) were also identified as being significantly increased in pregnant serum ExoE-EVs compared with NP. Due to the abundance of these X-chromosome miRNAs in placental tissue and trophoblast ExoE-EVs, it is likely that the ExoE-EVs containing mmu-miR-322-3p and mmu-miR-322-5p in pregnant mouse sera originated from the syncytial trophoblasts of the placenta. In addition to X-chromosome cluster miRNAs, mmu-miRNA-30d-5p was increased in syncytialized trophoblast compared to stem trophoblast ExoE-EVs. The observation that mmu-miR-30d-5p is increased in differentiated SynT ExoE-EVs is interesting as it has been confirmed to target and downregulate brain-derived neurotropic factor (BDNF) [52]. BDNF has been shown to be involved in regulation of trophoblast cell growth and survival [53, 54]. Therefore, the presence of these miRNAs within the pregnancy Exo-EVs may reflect the state of the placenta in terms of development and function in addition to having potential biologic impact on recipient cells.

The abundance of mmu-miR-292-3p and mmu-miR-183-5p increased with pregnancy, and were detected in trophoblast ExoE-EVs independent of differentiation state. This is somewhat surprising given that the mmu-miR-290 cluster of miRNAs was originally identified as being expressed exclusively by mouse embryonic stem cells (ESC) [55]. Consistent with the original report of mmu-miR-292-3p, more recent and comprehensive miRNA profiling detected mmu-miR-292-3p only in ESCs and teratocarcinoma [7]. Subsequent analysis of tissue expression of mmu-miR-183-5p showed high to moderate expression of mmu-miR-183-5p in ESC, embryo, and teratocarcinomas [7]. Our data suggests that trophoblasts are also a source. Additional investigation is required to determine the cellular source of pregnancy-associated circulating ExoE-EVs expressing miRNAs mmu-miR-292-3p and mmu-miR-183-5p, which could be either from the placenta or fetal tissue.

We were able to derive an enriched population of exosomes from the EVs from small blood volumes as well as obtain good quality RNA-seq data. Given that the EV material is not 100% exosomes, some of the identified RNA species may originate from other EVs or protein complexes contained within the plasma. Regardless of the exact origin, the fact that the miRNAs are increased or only present in pregnant plasma still highlights the role they may play in pregnancy. The RNA-seq data was limited in terms of the number and magnitude of expression difference that could be detected given the small sample size (n = 3). Utilizing the larger sample numbers for the qRT-PCR confirmation studies was valuable to validate not only the significant findings noted by RNA-seq but additional miRNAs that were not significant by RNA-seq. In the future, purifying trophoblast-derived exosomes from the pregnancy plasma would provide additional clarity to the origin of identified miRNAs.

Murine models are routinely used for the study of pregnancy and maternal-fetal tolerance, yet an important communication modality, placenta-derived EVs and specifically the miRNA cargo, has remained largely understudied. This study provides the first in-depth characterization of the RNA composition of *in vitro*-derived murine trophoblastic and pregnancy-associated exosome-enriched EVs. Importantly, this study identifies specific miRNAs that have significant roles in pregnancy physiology and potentially maternal-fetal tolerance and warrant further mechanistic studies. Determining the cargo of placental and pregnancy-specific ExoE-EVs and their predicted biological targets is important to advance our understanding of how these small membrane packages mediate cell-cell communication in pregnancy, as well as where these interactions may go awry in pregnancy-specific diseases.

### Future directions

Further investigation is necessary to determine the net biological effect of the multiple miRNAs contained within ExoE-EVs, on the vast number of each miRNA’s predicted targets. Additionally, confirmation of miRNA cargo in a pure exosome population would be ideal for validating our findings in the exosome-enriched EVs. For trophoblast-derived miRNAs, it will be important to consider both the local effects on reproductive and immune cells, as well as the systemic implications for maternal immune and endothelial cells.

## Supporting information

S1 Fig Differentiation of IV1 trophoblast stem cells (TSC) to syncytiotrophoblasts (SynT). Panel A shows bright field light microscopy images (20x) of IV1 cells in culture (CellStart with FGF4 and heparin) and then 7 days in the absence of FGF4 and heparin that leads to differentiation and cell fusion into SynT. Panel B Immunoblot of nuclear enriched fractions (NE-PER Nuclear and Cytoplasmic Extract Reagent from ThermoFisher) from undifferentiated IV1 TSC (U) and differentiated syncytiotrophoblasts (D) showing downregulation of stem cell markers CDX2 (abcam 76541), EOMES (abcam ab23345), and ELF5 (abcam ab104410), and alpha tubulin (aTUB; Sigma-Aldrich T9026) serves as loading control.
(TIF)

S2 Fig Pathways in cancer. (TIF)

S3 Fig MAPK signaling pathway. (TIF)

S4 Fig Focal adhesion pathway. (TIF)

S5 Fig Ubiquitin-mediated proteolysis. (TIF)

S6 Fig ARRIVE guidelines checklist. C57BL/6.J (B6) mice were cared for in a pathogen-free animal care facility at National Jewish Health in strict accordance with the recommendations in the Guide for the Care and Use of Laboratory Animals of the National Institutes of Health and Animal Research: Reporting of In Vivo Experiments (ARRIVE) guidelines.
(PDF)

S1 Table RNA-sequencing reads: Quality, trimming, and mapping. (TIF)

S2 Table X-chromosome miRNA predicted targets as identified by MultiMir analysis. Quality, trimming, and read mapping of sequenced RNAs.
(PDF)

S3 Table KEGG pathways predicted to be targeted by X-chromosome cluster microRNAs. (PDF)

S4 Table Quantitative RT-PCR primers. (TIF)
