# Supplementary figures and images for: Murine trophoblast-derived and pregnancy-associated exosome-enriched extracellular vesicle microRNAs: Implications for placenta driven effects on maternal physiology
Source: PLoS One. 2019 Feb 7;14(2):e0210675. doi: 10.1371/journal.pone.0210675 (PMC6366741; doi:10.1371/journal.pone.0210675)

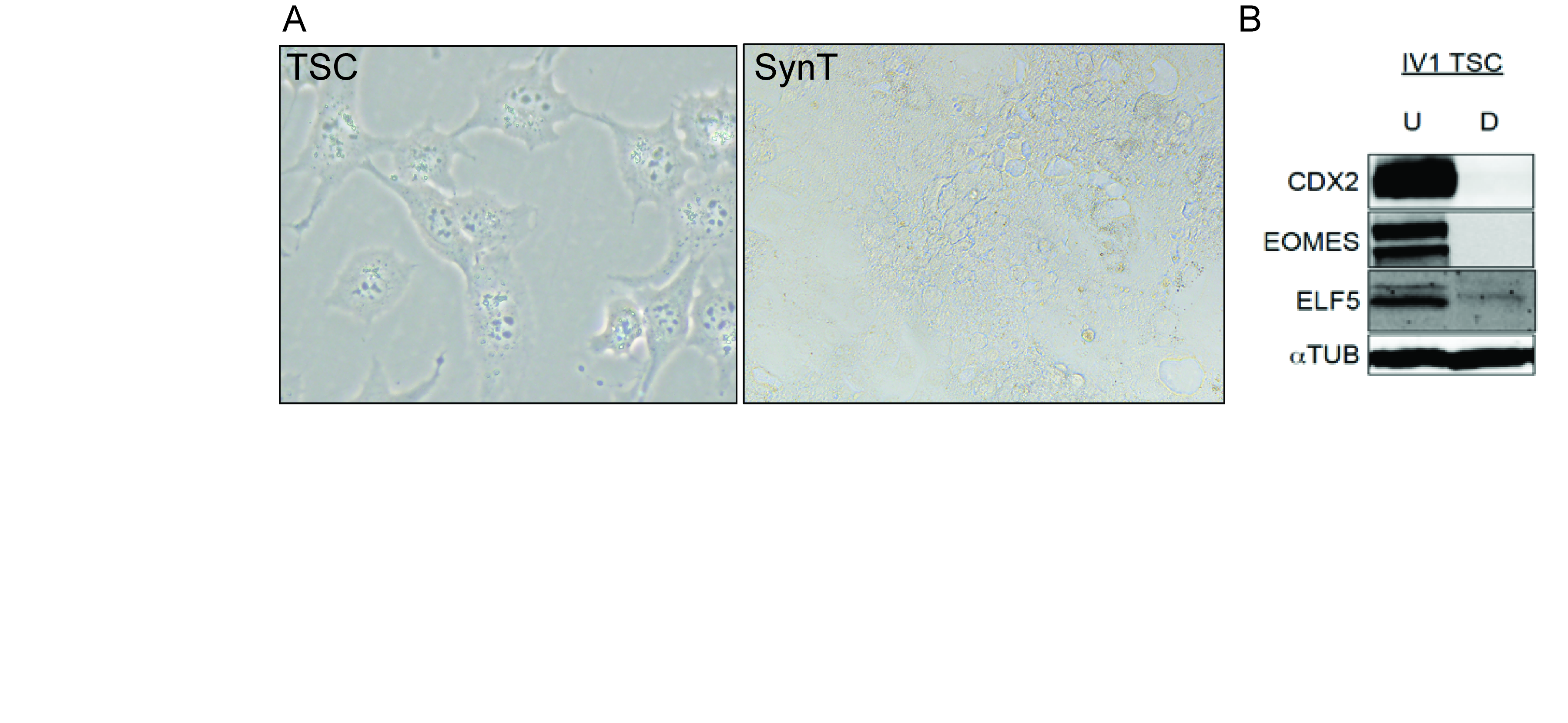

Supplement: S1 Fig — Panel A shows bright field light microscopy images (20x) of IV1 cells in culture (CellStart with FGF4 and heparin) and then 7 days in the absence of FGF4 and heparin that leads to differentiation and cell fusion into SynT. Panel B Immunoblot of nuclear enriched fractions (NE-PER Nuclear and Cytoplasmic Extract Reagent from ThermoFisher) from undifferentiated IV1 TSC (U) and differentiated syncytiotrophoblasts (D) showing downregulation of stem cell markers CDX2 (abcam 76541), EOMES (abcam ab23345), and ELF5 (abcam ab104410), and alpha tubulin (aTUB; Sigma-Aldrich T9026) serves as loading control. (TIF) [file pone.0210675.s001.tif]

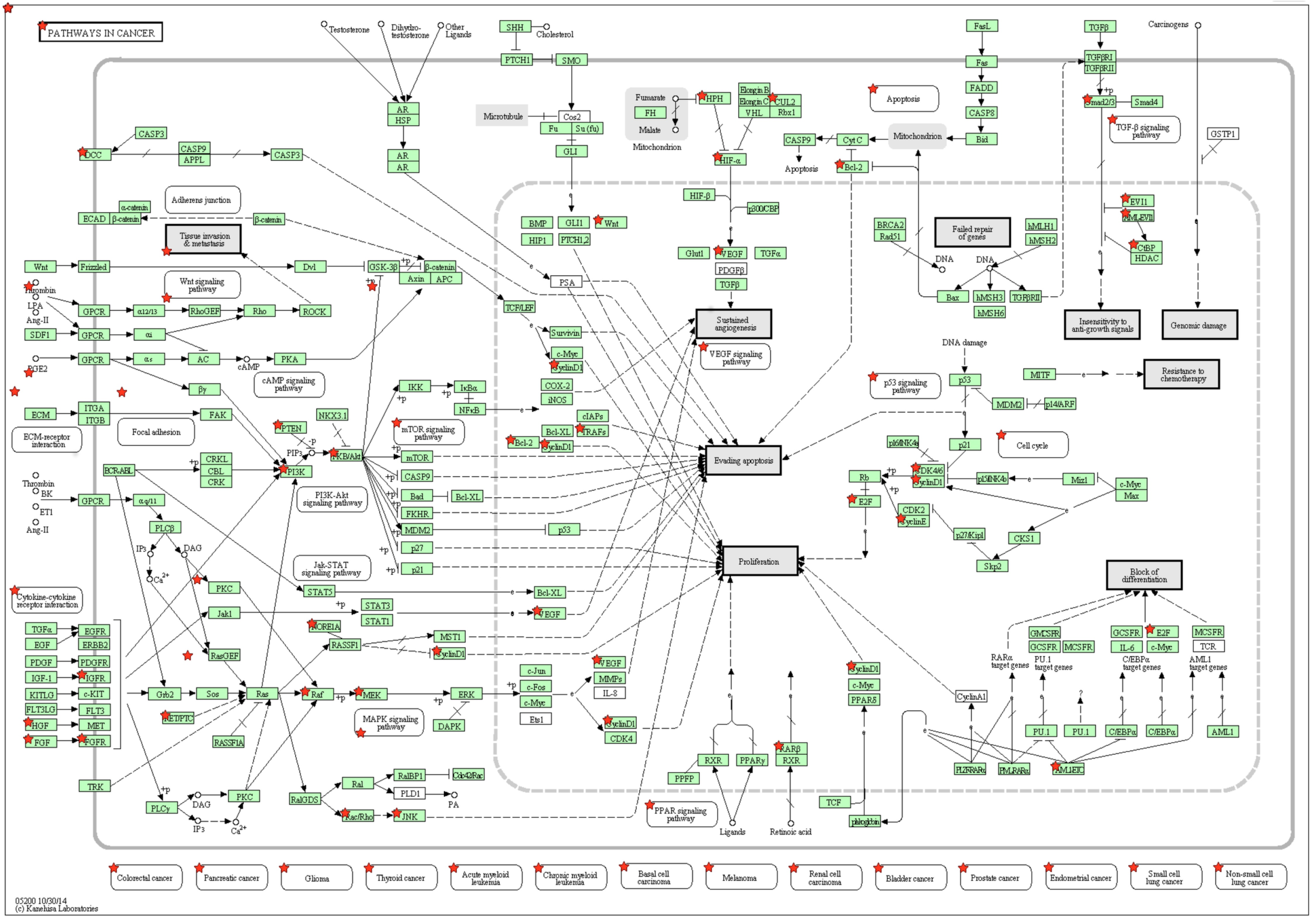

Supplement: S2 Fig — (TIF) [file pone.0210675.s002.tif]

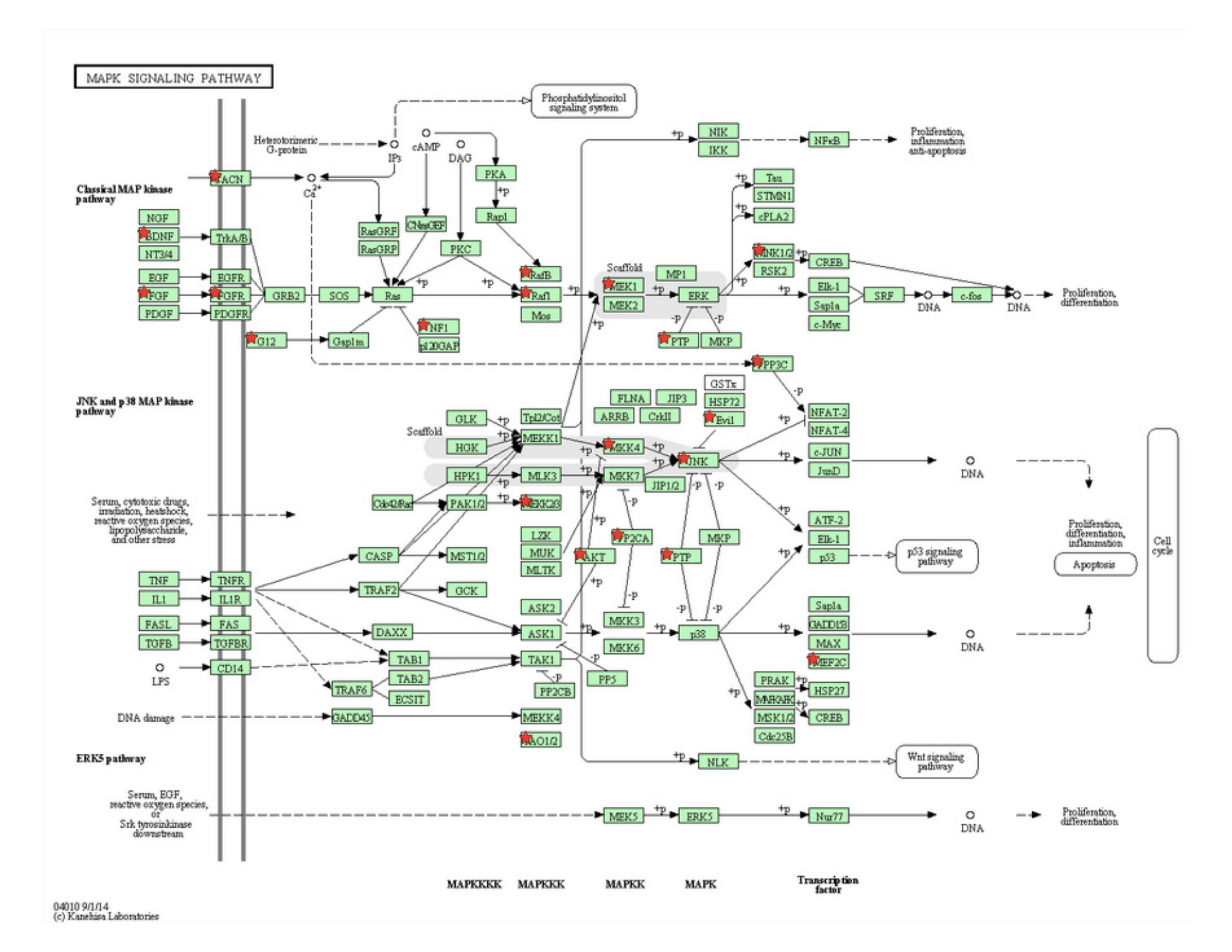

Supplement: S3 Fig — (TIF) [file pone.0210675.s003.tif]

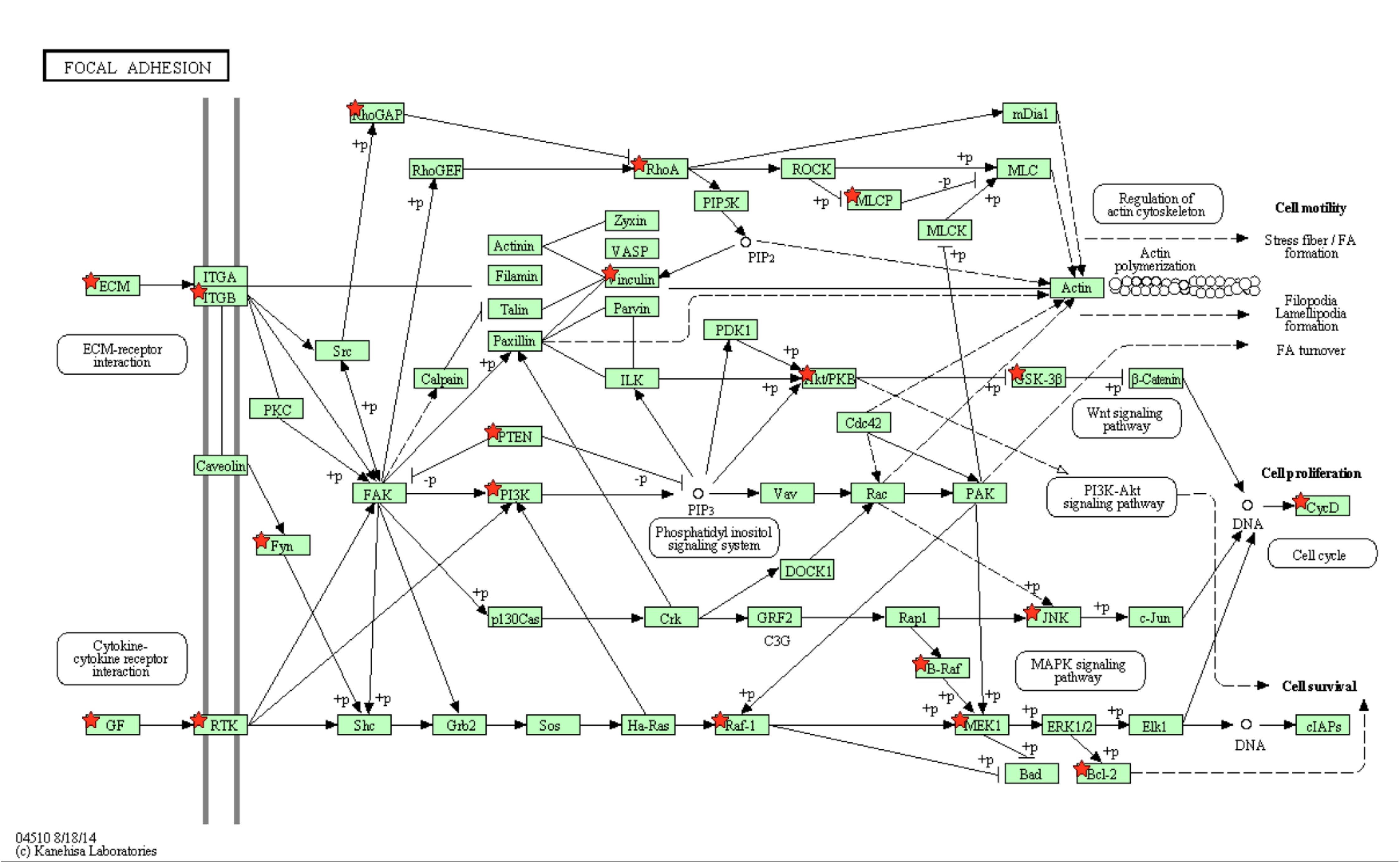

Supplement: S4 Fig — (TIF) [file pone.0210675.s004.tif]

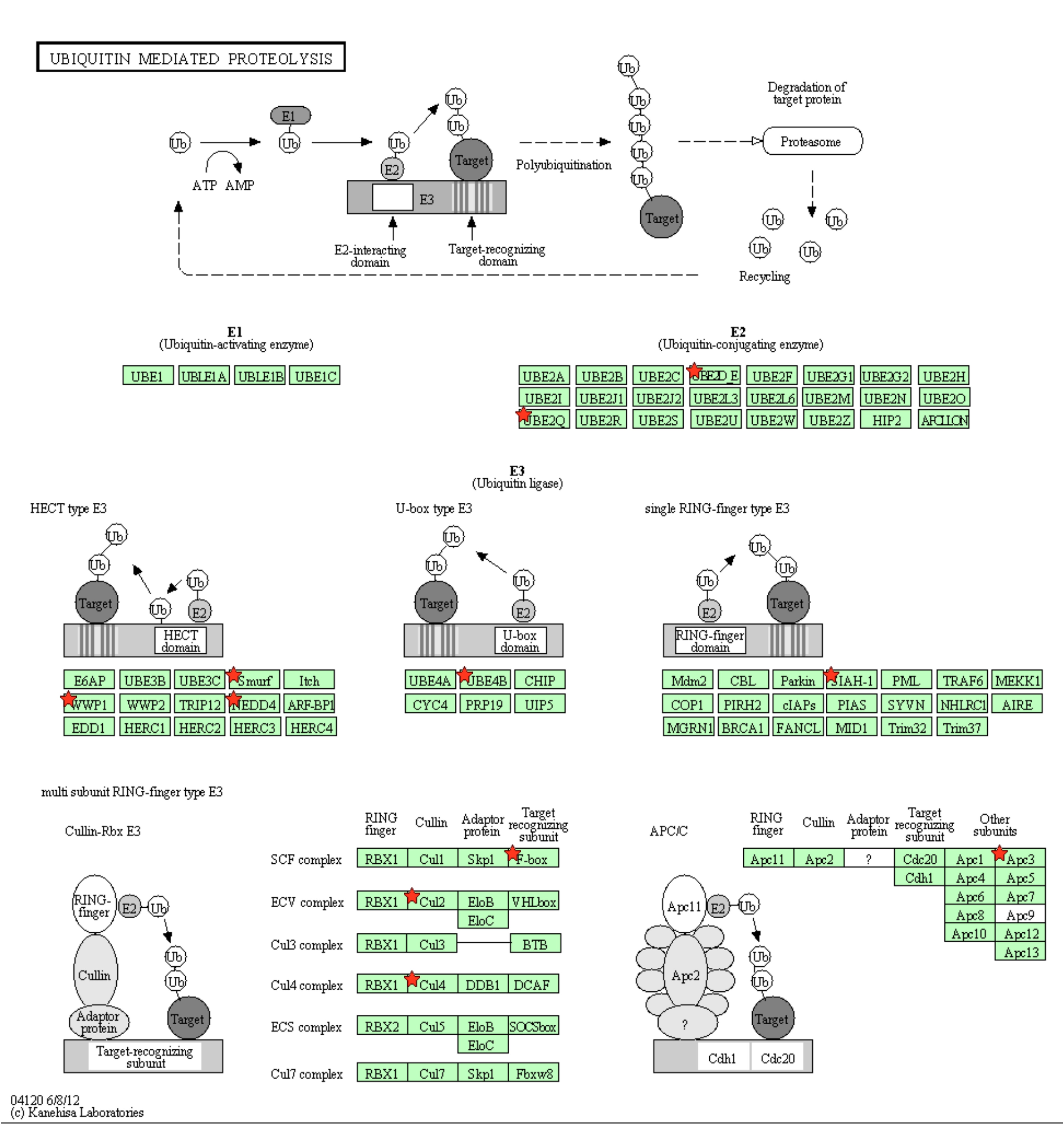

Supplement: S5 Fig — (TIF) [file pone.0210675.s005.tif]

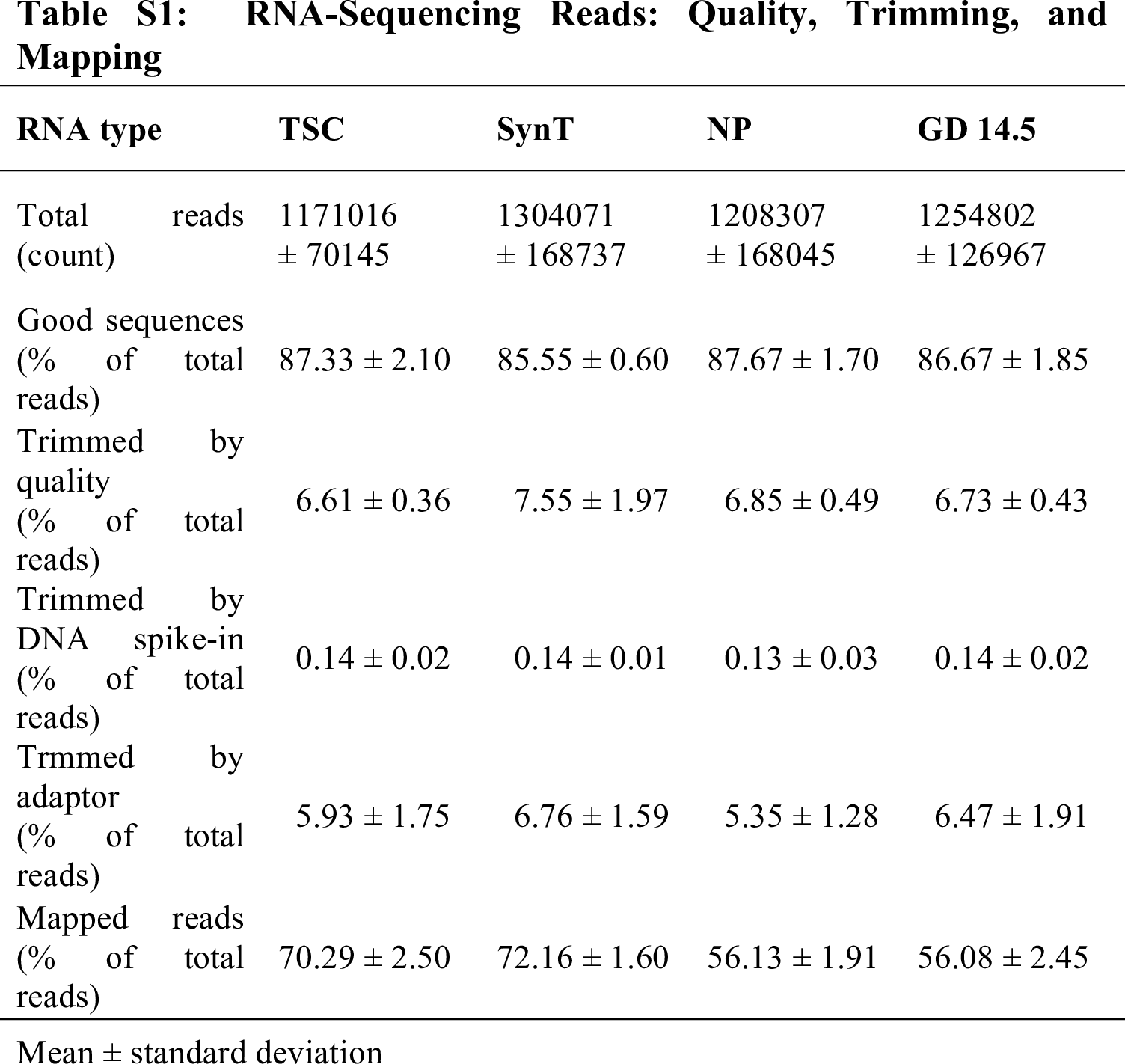

Supplement: S1 Table — (TIF) [file pone.0210675.s007.tif]

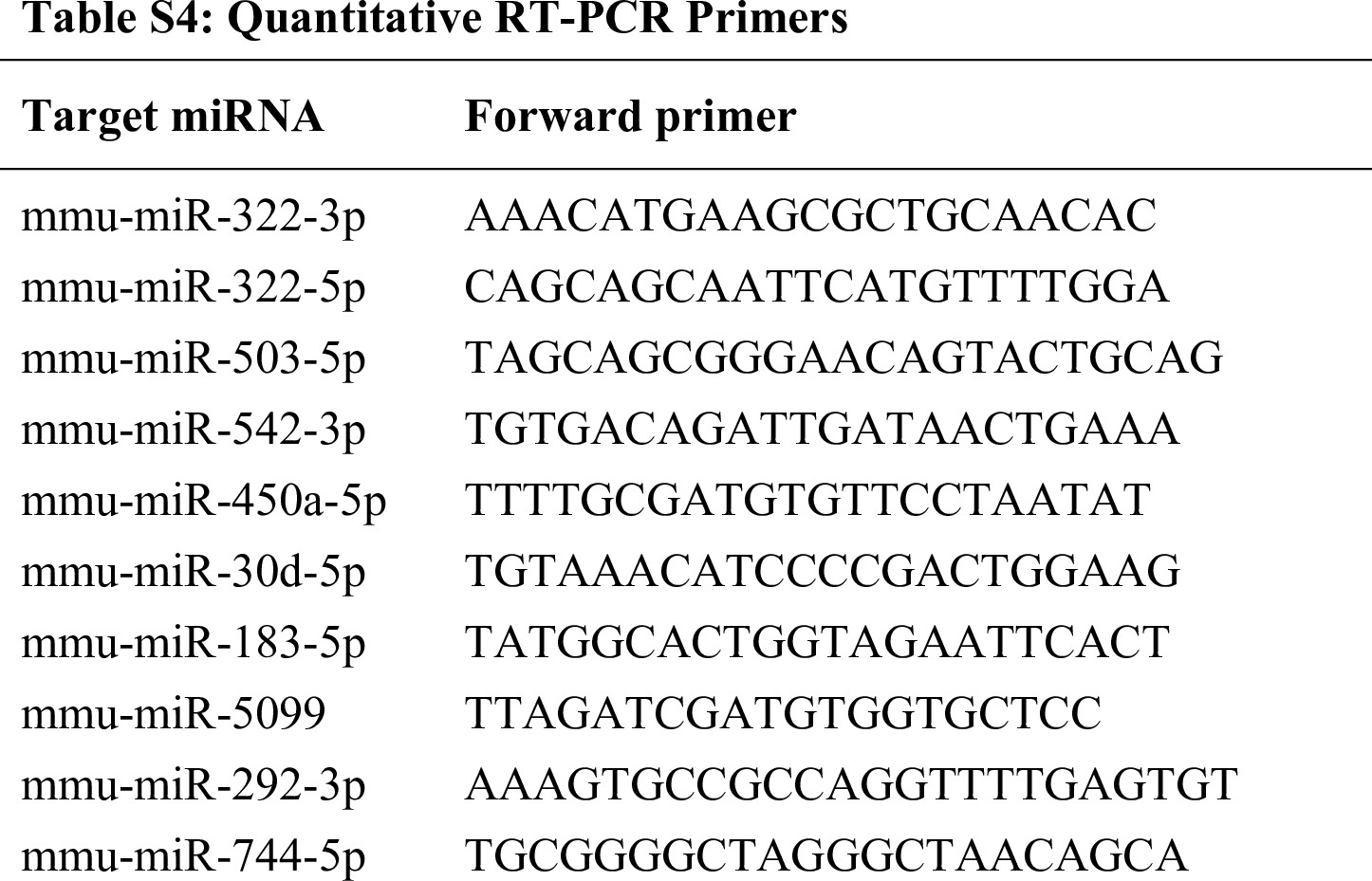

Supplement: S4 Table — (TIF) [file pone.0210675.s010.tif]
