# Supplementary material for: Murine trophoblast-derived and pregnancy-associated exosome-enriched extracellular vesicle microRNAs: Implications for placenta driven effects on maternal physiology
Source: PLoS One. 2019 Feb 7;14(2):e0210675. doi: 10.1371/journal.pone.0210675 (PMC6366741; doi:10.1371/journal.pone.0210675)
Supplement: S2 Table — Quality, trimming, and read mapping of sequenced RNAs. (PDF) [file pone.0210675.s008.pdf]

Table S2: X-Chromosome miRNA predicted targets as identified by MultiMir analysis

**Gene ID**	**Gene Name**	**322-3p**	**322-5p**	**503-5p**	**542-3p**
Sec24a	Sec24 related gene family, member A (S. cerevisiae)	1	1	1	0
Cpeb2	cytoplasmic polyadenylation element binding protein 2	1	1	1	0
Zbtb44	zinc finger and BTB domain containing 44	1	1	1	0
Tnrc6b	trinucleotide repeat containing 6b	1	1	1	0
Ccnd2	cyclin D2	0	1	1	0
Mnt	max binding protein	0	1	1	0
Raf1	v-raf-leukemia viral oncogene 1	0	1	1	0
Ddx3x	DEAD/H (Asp-Glu-Ala-Asp/His) box polypeptide 3, X-linked	0	1	1	0
Ccne1	cyclin E1	0	1	1	0
Dyrk1b	dual-specificity tyrosine-(Y)-phosphorylation regulated kinase 1b	0	1	1	0
Pnpla6	patatin-like phospholipase domain containing 6	0	1	1	0
Map2k1	mitogen-activated protein kinase kinase 1	0	1	1	0
Cbfa2t3	core-binding factor, runt domain, alpha subunit 2, translocated to, 3 (human)	0	1	1	0
Runx1t1	runt-related transcription factor 1; translocated to, 1 (cyclin D-related)	0	1	1	0
Kif5b	kinesin family member 5B	0	1	1	0
Cbx5	chromobox 5	0	1	0	1
Wnt3a	wingless-type MMTV integration site family, member 3A	0	1	1	0
E2f3	E2F transcription factor 3	0	1	1	0
Spag7	sperm associated antigen 7	0	1	1	0
Akt3	thymoma viral proto-oncogene 3	0	1	1	0
Myb	myeloblastosis oncogene	0	1	1	0
Cdk17	cyclin-dependent kinase 17	0	1	1	0
Actr2	ARP2 actin-related protein 2	0	1	1	0
Ap3d1	adaptor-related protein complex 3, delta 1 subunit	0	1	1	0
Pex13	peroxisomal biogenesis factor 13	1	1	0	0
Jade2	jade family PHD finger 2	0	1	0	1
Sypl	synaptophysin-like protein	1	1	0	0
Rab10	RAB10, member RAS oncogene family	0	1	1	0
Helz	helicase with zinc finger domain	0	1	1	0
Pafah1b1	platelet-activating factor acetylhydrolase, isoform 1b, subunit 1	0	1	1	0
Bmpr1a	bone morphogenetic protein receptor, type 1A	0	1	1	0
Kpna3	karyopherin (importin) alpha 3	1	0	1	0
Kif21a	kinesin family member 21A	0	1	1	0
Scn8a	sodium channel, voltage-gated, type VIII, alpha	0	1	1	0
Tle4	transducin-like enhancer of split 4, homolog of Drosophila E(spl)	0	1	1	0
Cdc37l1	cell division cycle 37-like 1	0	1	1	0
Arl2	ADP-ribosylation factor-like 2	0	1	1	0
Btrc	beta-transducin repeat containing protein	0	1	1	0
Zfhx4	zinc finger homeodomain 4	0	1	1	0
Smad7	SMAD family member 7	0	1	1	0
Mob4	MOB family member 4, phocein	0	1	1	0
Mgat4a	mannoside acetylglucosaminyltransferase 4, isoenzyme A	0	1	1	0
Speg	SPEG complex locus	0	1	0	1

Kif5c	kinesin family member 5C	0	1	1	0
Strbp	spermatid perinuclear RNA binding protein	1	1	0	0
Caprin1	cell cycle associated protein 1	1	1	0	0
Dclk1	doublecortin-like kinase 1	0	1	1	0
Ash1l	ash1 (absent, small, or homeotic)-like (Drosophila)	0	1	1	0
Tmem55a	transmembrane protein 55A	0	1	1	0
Epha7	Eph receptor A7	0	1	1	0
Pappa	pregnancy-associated plasma protein A	0	1	1	0
Cc2d1b	coiled-coil and C2 domain containing 1B	0	1	1	0
Zfp362	zinc finger protein 362	0	1	1	0
Ski	ski sarcoma viral oncogene homolog (avian)	0	1	1	0
Rasgef1b	RasGEF domain family, member 1B	0	1	1	0
Foxp2	forkhead box P2	0	1	1	0
Wipi2	WD repeat domain, phosphoinositide interacting 2	0	1	1	0
Wnt7a	wingless-type MMTV integration site family, member 7A	0	1	1	0
Ubfd1	ubiquitin family domain containing 1	0	1	1	0
Tbx22	T-box 22	1	1	0	0
Klhl2	kelch-like 2, Mayven	1	0	1	0
N4bp1	NEDD4 binding protein 1	0	1	1	0
Srpr	signal recognition particle receptor ('docking protein')	0	1	1	0
Chek1	checkpoint kinase 1	0	1	1	0
Kif23	kinesin family member 23	0	1	1	0
U2surp	U2 snRNP-associated SURP domain containing	0	1	0	1
Cdc25a	cell division cycle 25A	1	1	0	0
Rbm6	RNA binding motif protein 6	0	1	1	0
Prkar2a	protein kinase, cAMP dependent regulatory, type II alpha	0	1	1	0
1700025G04Rik	RIKEN cDNA 1700025G04 gene	0	1	1	0
Slc25a37	solute carrier family 25, member 37	0	1	1	0
Arhgap5	Rho GTPase activating protein 5	1	0	0	1
Prdm4	PR domain containing 4	0	1	1	0
Tmem55b	transmembrane protein 55b	0	1	1	0
Apln	apelin	0	1	1	0
Kctd8	potassium channel tetramerisation domain containing 8	1	1	0	0
Slc20a2	solute carrier family 20, member 2	0	1	1	0
Snrk	SNF related kinase	0	1	1	0
Sobp	sine oculis-binding protein homolog (Drosophila)	0	1	1	0
Ubn2	ubinuclein 2	0	1	1	0
Akap7	A kinase (PRKA) anchor protein 7	0	1	1	0
Cpeb3	cytoplasmic polyadenylation element binding protein 3	0	1	1	0
Cbx4	chromobox 4	1	1	0	0
Cacna2d1	calcium channel, voltage-dependent, alpha2/delta subunit 1	0	1	1	0
Bptf	bromodomain PHD finger transcription factor	0	1	1	0
Rfx3	regulatory factor X, 3 (influences HLA class II expression)	0	1	1	0
Tspyl2	TSPY-like 2	0	1	1	0
Pik3r1	phosphatidylinositol 3-kinase, regulatory subunit, polypeptide 1 (p85 alpha)	0	1	1	0
Kcnj2	potassium inwardly-rectifying channel, subfamily J, member 2	0	1	1	0
Atxn2	ataxin 2	0	1	1	0
Rsbn1	rosbin, round spermatid basic protein 1	0	1	1	0
Ubr3	ubiquitin protein ligase E3 component n-recognin 3	0	1	1	0

Crebrf	CREB3 regulatory factor	0	1	1	0
Lurap1l	leucine rich adaptor protein 1-like	0	1	1	0
Cdk5r1	cyclin-dependent kinase 5, regulatory subunit 1 (p35)	0	1	1	0
Tenm2	teneurin transmembrane protein 2	0	1	1	0
Omg	oligodendrocyte myelin glycoprotein	0	1	1	0
Rictor	RPTOR independent companion of MTOR, complex 2	0	1	1	0
Zfp217	zinc finger protein 217	0	1	1	0
Acvr2a	activin receptor IIA	0	1	1	0
Zfp622	zinc finger protein 622	0	1	1	0
Arhgap20	Rho GTPase activating protein 20	0	1	1	0
Ghr	growth hormone receptor	0	1	1	0
Socs6	suppressor of cytokine signaling 6	0	1	1	0
Bcl2	B cell leukemia/lymphoma 2	0	1	1	0
Pth	parathyroid hormone	0	1	1	0
Cd2ap	CD2-associated protein	0	1	1	0
Qk	quaking	0	1	1	0
Kif1b	kinesin family member 1B	1	1	0	0
Zbtb34	zinc finger and BTB domain containing 34	0	1	1	0
Trabd2b	TraB domain containing 2B	0	1	1	0
Mob3b	MOB kinase activator 3B	0	1	1	0
Fat3	FAT tumor suppressor homolog 3 (Drosophila)	0	1	0	1
Hiat1	hippocampus abundant gene transcript 1	1	0	0	1
Gnai3	guanine nucleotide binding protein (G protein), alpha inhibiting 3	0	1	0	0
Gna12	guanine nucleotide binding protein, alpha 12	0	1	0	0
Wap	whey acidic protein	0	1	0	0
Dlg3	discs, large homolog 3 (Drosophila)	0	1	0	0
Hoxa10	homeobox A10	0	1	0	0
Slc7a7	solute carrier family 7 (cationic amino acid transporter, y+ system), member 7	0	0	0	1
Luzp1	leucine zipper protein 1	0	1	0	0
Fbxo9	f-box protein 9	0	0	0	1
Kpnb1	karyopherin (importin) beta 1	0	1	0	0
Def8	differentially expressed in FDCP 8	0	1	0	0
Ell2	elongation factor RNA polymerase II 2	0	0	1	0
Hoxc8	homeobox C8	0	1	0	0
Chordc1	cysteine and histidine-rich domain (CHORD)-containing, zinc-binding protein 1	0	1	0	0
Sept7	septin 7	1	0	0	0
Kmt2a	lysine (K)-specific methyltransferase 2A	0	1	0	0
Sdf2	stromal cell derived factor 2	0	1	0	0
Mov10	Moloney leukemia virus 10	0	0	1	0
Gmpr2	guanosine monophosphate reductase 2	1	0	0	0
Braf	Braf transforming gene	0	1	0	0
Cdk12	cyclin-dependent kinase 12	0	1	0	0
Slc2a3	solute carrier family 2 (facilitated glucose transporter), member 3	0	1	0	0
Bicd1	bicaudal D homolog 1 (Drosophila)	0	1	0	0
Klc4	kinesin light chain 4	0	1	0	0
Man1a	mannosidase 1, alpha	1	0	0	0
Nfat5	nuclear factor of activated T cells 5	1	0	0	0

Ppfia3	protein tyrosine phosphatase, receptor type, f polypeptide (PTPRF), interacting protein (liprin), alpha 3	0	0	1	0
Il7r	interleukin 7 receptor	0	1	0	0
Clcn3	chloride channel 3	0	1	0	0
Pde1c	phosphodiesterase 1C	0	1	0	0
Eif2b2	eukaryotic translation initiation factor 2B, subunit 2 beta	0	1	0	0
Hdgf	hepatoma-derived growth factor	0	1	0	0
Chd5	chromodomain helicase DNA binding protein 5	0	1	0	0
Plekha8	pleckstrin homology domain containing, family A (phosphoinositide binding specific) member 8	0	0	0	1
Igf1r	insulin-like growth factor I receptor	0	0	1	0
Insr	insulin receptor	0	1	0	0
Mef2c	myocyte enhancer factor 2C	1	0	0	0
Zfp592	zinc finger protein 592	0	1	0	0
Tfap4	transcription factor AP4	0	1	0	0
Cd247	CD247 antigen	1	0	0	0
Rfx5	regulatory factor X, 5 (influences HLA class II expression)	0	1	0	0
Ankrd13d	ankyrin repeat domain 13 family, member D	0	1	0	0
Elovl1	elongation of very long chain fatty acids (FEN1/Elo2, SUR4/Elo3, yeast)-like 1	0	0	0	1
Ihh	Indian hedgehog	0	1	0	0
Usp19	ubiquitin specific peptidase 19	0	1	0	0
Ezh1	enhancer of zeste homolog 1 (Drosophila)	0	1	0	0
Pcdha11	protocadherin alpha 11	0	1	0	0
Homer1	homer homolog 1 (Drosophila)	0	0	1	0
Rhoa	ras homolog gene family, member A	1	0	0	0
Elavl2	ELAV (embryonic lethal, abnormal vision, Drosophila)-like 2 (Hu antigen B)	0	0	0	1
G0s2	G0/G1 switch gene 2	0	1	0	0
Pou6f1	POU domain, class 6, transcription factor 1	0	1	0	0
Nuak2	NUAK family, SNF1-like kinase, 2	0	1	0	0
Kdsr	3-ketodihydrosphingosine reductase	0	1	0	0
Gabra1	gamma-aminobutyric acid (GABA) A receptor, subunit alpha 1	0	0	1	0
Evi5	ecotropic viral integration site 5	0	1	0	0
Tmem167	transmembrane protein 167	0	1	0	0
Ttll5	tubulin tyrosine ligase-like family, member 5	0	1	0	0
Adgrl1	adhesion G protein-coupled receptor L1	0	1	0	0
Amotl1	angiomotin-like 1	0	1	0	0
Pten	phosphatase and tensin homolog	0	0	0	1
Rnf170	ring finger protein 170	0	0	0	1
Tmem87b	transmembrane protein 87B	0	1	0	0
Csf1	colony stimulating factor 1 (macrophage)	0	1	0	0
Tbp	TATA box binding protein	0	1	0	0
Dll1	delta-like 1 (Drosophila)	0	1	0	0
Tppp3	tubulin polymerization-promoting protein family member 3	0	1	0	0
Capza2	capping protein (actin filament) muscle Z-line, alpha 2	0	1	0	0
Lcorl	ligand dependent nuclear receptor corepressor-like	0	1	0	0
Sertad4	SERTA domain containing 4	1	0	0	0
Atp1b4	ATPase, (Na+)/K+ transporting, beta 4 polypeptide	0	1	0	0
Nup50	nucleoporin 50	0	1	0	0

Cntnap1	contactin associated protein-like 1	0	1	0	0
Taok1	TAO kinase 1	1	0	0	0
Cacnb4	calcium channel, voltage-dependent, beta 4 subunit	0	1	0	0
Rarb	retinoic acid receptor, beta	0	1	0	0
Suz12	suppressor of zeste 12 homolog (Drosophila)	0	1	0	0
Pitpna	phosphatidylinositol transfer protein, alpha	0	1	0	0
Med1	mediator complex subunit 1	0	1	0	0
Mtmr4	myotubularin related protein 4	0	1	0	0
Slc13a3	solute carrier family 13 (sodium-dependent dicarboxylate transporter), member 3	0	1	0	0
Gabarap	gamma-aminobutyric acid receptor associated protein	0	0	0	1
G3bp1	GTPase activating protein (SH3 domain) binding protein 1	1	0	0	0
Rad51d	RAD51 homolog D	0	1	0	0
Ywhah	tyrosine 3-monooxygenase/tryptophan 5-monooxygenase activation protein, eta polypeptide	0	1	0	0
Zfp687	zinc finger protein 687	0	1	0	0
Trip10	thyroid hormone receptor interactor 10	0	1	0	0
Pcmt1	protein-L-isoaspartate (D-aspartate) O-methyltransferase 1	0	1	0	0
Nr2e1	nuclear receptor subfamily 2, group E, member 1	0	1	0	0
Rev3l	REV3-like, catalytic subunit of DNA polymerase zeta RAD54 like (S. cerevisiae)	1	0	0	0
Fyn	Fyn proto-oncogene	1	0	0	0
Rspo3	R-spondin 3 homolog (Xenopus laevis)	0	1	0	0
Ppp1r12a	protein phosphatase 1, regulatory (inhibitor) subunit 12A	1	0	0	0
Sgk1	serum/glucocorticoid regulated kinase 1	0	1	0	0
Map7	microtubule-associated protein 7	0	1	0	0
Ptprr	protein tyrosine phosphatase, receptor type, R	0	1	0	0
E2f7	E2F transcription factor 7	0	1	0	0
Nfyb	nuclear transcription factor-Y beta	1	0	0	0
Wdr82	WD repeat domain containing 82	0	1	0	0
Slc36a1	solute carrier family 36 (proton/amino acid symporter), member 1	0	1	0	0
Fbxw11	F-box and WD-40 domain protein 11	1	0	0	0
Sptbn1	spectrin beta, non-erythrocytic 1	1	0	0	0
Btg2	B cell translocation gene 2, anti-proliferative	0	1	0	0
Ppm1d	protein phosphatase 1D magnesium-dependent, delta isoform	0	1	0	0
Taf15	TAF15 RNA polymerase II, TATA box binding protein (TBP)-associated factor	0	1	0	0
Cdc27	cell division cycle 27	0	0	1	0
Map3k3	mitogen-activated protein kinase kinase kinase 3	0	1	0	0
Nf1	neurofibromatosis 1	0	1	0	0
Gga3	golgi associated, gamma adaptin ear containing, ARF binding protein 3	0	1	0	0
Cluh	clustered mitochondria (cluA/CLU1) homolog	0	1	0	0
Acox1	acyl-Coenzyme A oxidase 1, palmitoyl	0	1	0	0
Kif1c	kinesin family member 1C	0	0	1	0
Cpd	carboxypeptidase D	0	1	0	0
Fbxl20	F-box and leucine-rich repeat protein 20	0	1	0	0
Ctage5	CTAGE family, member 5	1	0	0	0
Nova1	neuro-oncological ventral antigen 1	1	0	0	0

Ppm1a	protein phosphatase 1A, magnesium dependent, alpha isoform	1	0	0	0
Hif1a	hypoxia inducible factor 1, alpha subunit	1	0	0	0
Vti1b	vesicle transport through interaction with t-SNAREs 1B	0	1	0	0
Tshz3	teashirt zinc finger family member 3	1	0	0	0
Traf3	TNF receptor-associated factor 3	0	1	0	0
Lrrc16a	leucine rich repeat containing 16A	1	0	0	0
Prpf4b	PRP4 pre-mRNA processing factor 4 homolog B (yeast)	1	0	0	0
Sptlc1	serine palmitoyltransferase, long chain base subunit 1	0	1	0	0
Drd1	dopamine receptor D1	0	1	0	0
Smad5	SMAD family member 5	0	1	0	0
Mctp1	multiple C2 domains, transmembrane 1	1	0	0	0
Btf3	basic transcription factor 3	0	0	1	0
Col4a3bp	collagen, type IV, alpha 3 (Goodpasture antigen) binding protein	0	1	0	0
Scamp1	secretory carrier membrane protein 1	1	0	0	0
Slc4a7	solute carrier family 4, sodium bicarbonate cotransporter, member 7	0	1	0	0
Ppap2a	phosphatidic acid phosphatase type 2A	0	1	0	0
Fst	follistatin	1	0	0	0
Ube2e1	ubiquitin-conjugating enzyme E2E 1	0	0	0	1
Glud1	glutamate dehydrogenase 1	0	1	0	0
Ppp3cb	protein phosphatase 3, catalytic subunit, beta isoform	0	1	0	0
Vcl	vinculin	1	0	0	0
Mapk1ip1l	mitogen-activated protein kinase 1 interacting protein 1-like	0	1	0	0
Mapk8	mitogen-activated protein kinase 8	0	1	0	0
Zmym2	zinc finger, MYM-type 2	0	1	0	0
Dcp1a	DCP1 decapping enzyme homolog A (S. cerevisiae)	0	1	0	0
Hmbox1	homeobox containing 1	0	1	0	0
Fgf9	fibroblast growth factor 9	0	1	0	0
Extl3	exostoses (multiple)-like 3	0	0	1	0
Wnt5a	wingless-type MMTV integration site family, member 5A	1	0	0	0
Lcp1	lymphocyte cytosolic protein 1	1	0	0	0
Tsc22d1	TSC22 domain family, member 1	0	0	0	1
Xpo7	exportin 7	0	1	0	0
Dcaf11	DDB1 and CUL4 associated factor 11	1	0	0	0
Zhx1	zinc fingers and homeoboxes 1	0	1	0	0
Fam49b	family with sequence similarity 49, member B	1	0	0	0
Desi1	desumoylating isopeptidase 1	0	1	0	0
1810013L24Rik	RIKEN cDNA 1810013L24 gene	0	1	0	0
Atp13a3	ATPase type 13A3	0	1	0	0
Alcam	activated leukocyte cell adhesion molecule	0	0	0	1
Ube2v2	ubiquitin-conjugating enzyme E2 variant 2	1	0	0	0
Sidt1	SID1 transmembrane family, member 1	0	1	0	0
Usp7	ubiquitin specific peptidase 7	0	1	0	0
Gsk3b	glycogen synthase kinase 3 beta	0	1	0	0
Usp25	ubiquitin specific peptidase 25	1	0	0	0
App	amyloid beta (A4) precursor protein	0	1	0	0
Adamts5	a disintegrin-like and metallopeptidase (reprolysin type) with thrombospondin type 1 motif, 5 (aggrecanase-2)	0	1	0	0

Dyrk1a	dual-specificity tyrosine-(Y)-phosphorylation regulated kinase 1a	0	1	0	0
Kpna1	karyopherin (importin) alpha 1	0	1	0	0
Son	Son DNA binding protein	0	1	0	0
Synj1	synaptojanin 1	0	1	0	0
Slc4a8	solute carrier family 4 (anion exchanger), member 8	0	1	0	0
Tarbp2	TAR (HIV) RNA binding protein 2	0	1	0	0
Sncg	synuclein, gamma	0	1	0	0
Akirin1	akirin 1	0	0	1	0
Pisd	phosphatidylserine decarboxylase	0	1	0	0
Igf2r	insulin-like growth factor 2 receptor	0	1	0	0
Vegfa	vascular endothelial growth factor A	0	1	0	0
Ptk7	PTK7 protein tyrosine kinase 7	0	0	1	0
Pim1	proviral integration site 1	0	1	0	0
Anks1	ankyrin repeat and SAM domain containing 1	0	0	1	0
Cul2	cullin 2	0	1	0	0
Syt4	synaptotagmin IV	0	1	0	0
Rnf138	ring finger protein 138	0	1	0	0
Sall3	sal-like 3 (Drosophila)	0	0	1	0
Nedd4l	neural precursor cell expressed, developmentally down-regulated gene 4-like	1	0	0	0
Slc12a2	solute carrier family 12, member 2	0	1	0	0
Rtn3	reticulon 3	0	1	0	0
Otub1	OTU domain, ubiquitin aldehyde binding 1	0	1	0	0
Pcgf5	polycomb group ring finger 5	1	0	0	0
Rad9a	RAD9 homolog A	0	1	0	0
Gldc	glycine decarboxylase	0	0	0	1
Peli3	pellino 3	0	1	0	0
Esrra	estrogen related receptor, alpha	0	1	0	0
Shoc2	soc-2 (suppressor of clear) homolog (C. elegans)	0	1	0	0
Eif3a	eukaryotic translation initiation factor 3, subunit A	0	1	0	0
Arl3	ADP-ribosylation factor-like 3	0	1	0	0
Arhgdia	Rho GDP dissociation inhibitor (GDI) alpha	0	1	0	0
Entpd7	ectonucleoside triphosphate diphosphohydrolase 7	0	1	0	0
Arih1	ariadne ubiquitin-conjugating enzyme E2 binding protein homolog 1 (Drosophila)	0	1	0	0
Itgb8	integrin beta 8	0	1	0	0
Sp4	trans-acting transcription factor 4	1	0	0	0
Aatk	apoptosis-associated tyrosine kinase	0	1	0	0
Hdhd2	haloacid dehalogenase-like hydrolase domain containing 2	1	0	0	0
Clns1a	chloride channel, nucleotide-sensitive, 1A	0	0	0	1
Cpeb1	cytoplasmic polyadenylation element binding protein 1	0	1	0	0
Cnksr2	connector enhancer of kinase suppressor of Ras 2	0	1	0	0
Hspa4l	heat shock protein 4 like	0	1	0	0
Pgm2	phosphoglucomutase 2	0	1	0	0
Gria4	glutamate receptor, ionotropic, AMPA4 (alpha 4)	0	0	0	1
Mybl1	myeloblastosis oncogene-like 1	1	0	0	0
Eya1	eyes absent 1 homolog (Drosophila)	0	1	0	0
Tram1	translocating chain-associating membrane protein 1	0	1	0	0
Slc39a10	solute carrier family 39 (zinc transporter), member 10	0	1	0	0
Cd28	CD28 antigen	0	1	0	0
Eif5b	eukaryotic translation initiation factor 5B	0	1	0	0

Tmeff2	transmembrane protein with EGF-like and two follistatin-like domains 2	1	0	0	0
Dst	dystonin	1	0	0	0
Fam135a	family with sequence similarity 135, member A	0	0	1	0
Agfg1	ArfGAP with FG repeats 1	1	0	0	0
Tuba4a	tubulin, alpha 4A	0	1	0	0
Htr4	5 hydroxytryptamine (serotonin) receptor 4	0	1	0	0
Pam	peptidylglycine alpha-amidating monooxygenase	0	0	1	0
Ccnt2	cyclin T2	0	1	0	0
Tsn	translin	1	0	0	0
Rassf5	Ras association (RalGDS/AF-6) domain family member 5	0	1	0	0
Nucks1	nuclear casein kinase and cyclin-dependent kinase substrate 1	0	1	0	0
Xpr1	xenotropic and polytropic retrovirus receptor 1	0	1	0	0
Lamc1	laminin, gamma 1	0	1	0	0
Desi2	desumoylating isopeptidase 2	1	0	0	0
Cfap45	cilia and flagella associated protein 45	0	1	0	0
Abl2	v-abl Abelson murine leukemia viral oncogene 2 (arg, Abelson-related gene)	0	1	0	0
Esrrg	estrogen-related receptor gamma	0	1	0	0
Tmem206	transmembrane protein 206	0	1	0	0
Plxna2	plexin A2	0	1	0	0
Hsd17b7	hydroxysteroid (17-beta) dehydrogenase 7	1	0	0	0
Ppp6c	protein phosphatase 6, catalytic subunit	0	1	0	0
Golga1	golgi autoantigen, golgin subfamily a, 1	0	1	0	0
Zbtb43	zinc finger and BTB domain containing 43	0	1	0	0
Olfm1	olfactomedin 1	1	0	0	0
Dolpp1	dolichyl pyrophosphate phosphatase 1	0	1	0	0
Phf19	PHD finger protein 19	0	1	0	0
Lhx6	LIM homeobox protein 6	0	1	0	0
Camsap1	calmodulin regulated spectrin-associated protein 1	0	1	0	0
Lhx3	LIM homeobox protein 3	0	1	0	0
Lrp2	low density lipoprotein receptor-related protein 2	0	1	0	0
Sp3	trans-acting transcription factor 3	1	0	0	0
Ttc17	tetratricopeptide repeat domain 17	0	0	1	0
Sema6d	sema domain, transmembrane domain (TM), and cytoplasmic domain, (semaphorin) 6D	0	1	0	0
Cops2	COP9 (constitutive photomorphogenic) homolog, subunit 2 (Arabidopsis thaliana)	0	1	0	0
Arfgap2	ADP-ribosylation factor GTPase activating protein 2	0	1	0	0
Mkks	McKusick-Kaufman syndrome	0	1	0	0
Mtch2	mitochondrial carrier homolog 2 (C. elegans)	0	1	0	0
Dll4	delta-like 4 (Drosophila)	0	1	0	0
1700037H04Rik	RIKEN cDNA 1700037H04 gene	0	1	0	0
Polr3f	polymerase (RNA) III (DNA directed) polypeptide F	0	1	0	0
Bpifa1	BPI fold containing family A, member 1	0	1	0	0
Bpifa5	BPI fold containing family A, member 5	0	1	0	0
Phactr3	phosphatase and actin regulator 3	1	0	0	0
Snx16	sorting nexin 16	0	1	0	0
Sall4	sal-like 4 (Drosophila)	0	1	0	0
Tbl1xr1	transducin (beta)-like 1X-linked receptor 1	0	1	0	0
Ttc14	tetratricopeptide repeat domain 14	0	1	0	0

Mecom	MDS1 and EVI1 complex locus	1	0	0	0
Sec62	SEC62 homolog (S. cerevisiae)	0	1	0	0
Slc7a11	solute carrier family 7 (cationic amino acid transporter, y+ system), member 11	0	0	0	1
Nbea	neurobeachin	1	0	0	0
Tsc22d2	TSC22 domain family, member 2	1	0	0	0
Kcnab1	potassium voltage-gated channel, shaker-related subfamily, beta member 1	0	1	0	0
Pdcd10	programmed cell death 10	1	0	0	0
Syt6	synaptotagmin VI	0	0	0	1
Stxbp3a	syntaxin binding protein 3A	0	1	0	0
Plrg1	pleiotropic regulator 1, PRL1 homolog (Arabidopsis)	0	1	0	0
Gucy1b3	guanylate cyclase 1, soluble, beta 3	1	0	0	0
Fubp1	far upstream element (FUSE) binding protein 1	1	0	0	0
Fbxw7	F-box and WD-40 domain protein 7	0	1	0	0
Tspan5	tetraspanin 5	0	1	0	0
Cth	cystathionase (cystathionine gamma-lyase)	1	0	0	0
Col24a1	collagen, type XXIV, alpha 1	0	1	0	0
Asph	aspartate-beta-hydroxylase	1	0	0	0
Calb1	calbindin 1	0	0	0	1
Tmem68	transmembrane protein 68	1	0	0	0
Pnisr	PNN interacting serine/arginine-rich	0	1	0	0
Rragd	Ras-related GTP binding D	1	0	0	0
Ptbp3	polypyrimidine tract binding protein 3	1	0	0	0
Mpdz	multiple PDZ domain protein	1	0	0	0
Rad23b	RAD23b homolog (S. cerevisiae)	0	1	0	0
Rgp1	RGP1 retrograde golgi transport homolog (S. cerevisiae)	0	1	0	0
Reck	reversion-inducing-cysteine-rich protein with kazal motifs	0	1	0	0
Ak4	adenylate kinase 4	0	1	0	0
Elavl4	ELAV (embryonic lethal, abnormal vision, Drosophila)-like 4 (Hu antigen D)	1	0	0	0
Caap1	caspase activity and apoptosis inhibitor 1	1	0	0	0
Lypla2	lysophospholipase 2	0	1	0	0
Mknk1	MAP kinase-interacting serine/threonine kinase 1	0	1	0	0
Hgf	hepatocyte growth factor	0	1	0	0
Sema3a	sema domain, immunoglobulin domain (Ig), short basic domain, secreted, (semaphorin) 3A	0	1	0	0
Ube4b	ubiquitination factor E4B	0	1	0	0
Mfn2	mitofusin 2	0	1	0	0
Nsg1	neuron specific gene family member 1	0	1	0	0
Fosl2	fos-like antigen 2	0	1	0	0
Nrbp1	nuclear receptor binding protein 1	0	1	0	0
Ppargc1a	peroxisome proliferative activated receptor, gamma, coactivator 1 alpha	0	1	0	0
Sel1l3	sel-1 suppressor of lin-12-like 3 (C. elegans)	0	1	0	0
Tbc1d14	TBC1 domain family, member 14	1	0	0	0
Ugdh	UDP-glucose dehydrogenase	1	0	0	0
Apbb2	amyloid beta (A4) precursor protein-binding, family B, member 2	0	0	0	1
Slc30a9	solute carrier family 30 (zinc transporter), member 9	1	0	0	0
Clock	circadian locomotor output cycles kaput	0	1	0	0
Mtf2	metal response element binding transcription factor 2	0	0	0	1

Fam69a	family with sequence similarity 69, member A	0	1	0	0
Tgfbr3	transforming growth factor, beta receptor III	0	1	0	0
Hnrnpdl	heterogeneous nuclear ribonucleoprotein D-like	1	0	0	0
Ereg	epiregulin	1	0	0	0
Tmed2	transmembrane emp24 domain trafficking protein 2	1	0	0	0
Sfswap	splicing factor, suppressor of white-apricot homolog (Drosophila)	1	0	0	0
Pdap1	PDGFA associated protein 1	0	1	0	0
Ndufa4	NADH dehydrogenase (ubiquinone) 1 alpha subcomplex, 4	1	0	0	0
Usp12	ubiquitin specific peptidase 12	0	1	0	0
Zkscan1	zinc finger with KRAB and SCAN domains 1	0	1	0	0
Dync1i1	dynein cytoplasmic 1 intermediate chain 1	0	1	0	0
Plxna4	plexin A4	0	1	0	0
Ahcyl2	S-adenosylhomocysteine hydrolase-like 2	0	1	0	0
Avl9	AVL9 homolog (S. cerevisiase)	0	1	0	0
4921507P07Rik	RIKEN cDNA 4921507P07 gene	1	0	0	0
Zfml	zinc finger, matrin-like	1	0	0	0
Lrig1	leucine-rich repeats and immunoglobulin-like domains 1	0	1	0	0
Nup210	nucleoporin 210	0	1	0	0
Itpr1	inositol 1,4,5-trisphosphate receptor 1	0	1	0	0
Ret	ret proto-oncogene	0	1	0	0
Tmcc1	transmembrane and coiled coil domains 1	0	1	0	0
Lrp6	low density lipoprotein receptor-related protein 6	0	1	0	0
Wbp11	WW domain binding protein 11	0	1	0	0
Aebp2	AE binding protein 2	1	0	0	0
Rassf8	Ras association (RalGDS/AF-6) domain family (N-terminal) member 8	0	1	0	0
Etnk1	ethanolamine kinase 1	0	1	0	0
Vamp1	vesicle-associated membrane protein 1	0	1	0	0
Crtc3	CREB regulated transcription coactivator 3	0	1	0	0
Vps33b	vacuolar protein sorting 33B (yeast)	0	1	0	0
Rab38	RAB38, member RAS oncogene family	0	0	0	1
Pik3c2a	phosphatidylinositol 3-kinase, C2 domain containing, alpha polypeptide	0	0	1	0
Ipo5	importin 5	1	0	0	0
Arhgap17	Rho GTPase activating protein 17	1	0	0	0
Rbbp6	retinoblastoma binding protein 6	0	1	0	0
Gga2	golgi associated, gamma adaptin ear containing, ARF binding protein 2	0	1	0	0
Stim1	stromal interaction molecule 1	0	1	0	0
Usp9x	ubiquitin specific peptidase 9, X chromosome	0	1	0	0
Cask	calcium/calmodulin-dependent serine protein kinase (MAGUK family)	0	1	0	0
Swap70	SWA-70 protein	0	1	0	0
Wee1	WEE 1 homolog 1 (S. pombe)	0	1	0	0
Zfp300	zinc finger protein 300	0	1	0	0
Slc6a14	solute carrier family 6 (neurotransmitter transporter), member 14	1	0	0	0
Cul4b	cullin 4B	1	0	0	0
Smarca1	SWI/SNF related, matrix associated, actin dependent regulator of chromatin, subfamily a, member 1	0	0	1	0

Gpc4	glypican 4	1	0	0	0
Fgf13	fibroblast growth factor 13	0	0	0	1
Dynlt3	dynein light chain Tctex-type 3	0	1	0	0
Tnmd	tenomodulin	1	0	0	0
Acsl4	acyl-CoA synthetase long-chain family member 4	0	1	0	0
Lrch2	leucine-rich repeats and calponin homology (CH) domain containing 2	1	0	0	0
Chic1	cysteine-rich hydrophobic domain 1	0	1	0	0
Gpm6b	glycoprotein m6b	1	0	0	0
Zfp275	zinc finger protein 275	0	1	0	0
Mpp1	membrane protein, palmitoylated	0	1	0	0
Tpte	transmembrane phosphatase with tensin homology	1	0	0	0
Slit2	slit homolog 2 (Drosophila)	0	1	0	0
Fgfr1	fibroblast growth factor receptor 1	0	1	0	0
Slc7a2	solute carrier family 7 (cationic amino acid transporter, y+ system), member 2	0	1	0	0
Fgf20	fibroblast growth factor 20	0	1	0	0
Sall1	sal-like 1 (Drosophila)	0	1	0	0
Pou4f2	POU domain, class 4, transcription factor 2	0	1	0	0
Dnaja2	DnaJ (Hsp40) homolog, subfamily A, member 2	0	0	1	0
Phlpp2	PH domain and leucine rich repeat protein phosphatase 2	0	1	0	0
Cx3cl1	chemokine (C-X3-C motif) ligand 1	0	1	0	0
Nfatc3	nuclear factor of activated T cells, cytoplasmic, calcineurin dependent 3	0	1	0	0
Pla2g15	phospholipase A2, group XV	0	1	0	0
Vps26b	vacuolar protein sorting 26 homolog B (yeast)	1	0	0	0
Usp2	ubiquitin specific peptidase 2	0	0	1	0
Pvrl1	poliovirus receptor-related 1	0	1	0	0
Ppp2r1b	protein phosphatase 2, regulatory subunit A, beta	0	1	0	0
Cadm1	cell adhesion molecule 1	0	1	0	0
Bace1	beta-site APP cleaving enzyme 1	0	1	0	0
Ddx25	DEAD (Asp-Glu-Ala-Asp) box polypeptide 25	0	0	0	1
Nlrx1	NLR family member X1	0	1	0	0
Abcg4	ATP-binding cassette, sub-family G (WHITE), member 4	0	1	0	0
Rnf111	ring finger 111	0	1	0	0
Phip	pleckstrin homology domain interacting protein	0	1	0	0
Col12a1	collagen, type XII, alpha 1	0	1	0	0
Neo1	neogenin	1	0	0	0
Smad3	SMAD family member 3	0	1	0	0
Lrrfip2	leucine rich repeat (in FLII) interacting protein 2	0	1	0	0
Arpp21	cyclic AMP-regulated phosphoprotein, 21	0	1	0	0
Pdcd6ip	programmed cell death 6 interacting protein	0	1	0	0
Srprb	signal recognition particle receptor, B subunit	0	1	0	0
Ip6k1	inositol hexaphosphate kinase 1	0	0	1	0
Nckipsd	NCK interacting protein with SH3 domain	0	1	0	0
Crebl2	cAMP responsive element binding protein-like 2	0	1	0	0
Kank1	KN motif and ankyrin repeat domains 1	0	0	1	0
Abtb2	ankyrin repeat and BTB (POZ) domain containing 2	0	1	0	0
Snx33	sorting nexin 33	0	1	0	0
Ank2	ankyrin 2, brain	0	1	0	0
Fbxo21	F-box protein 21	0	1	0	0
Lrig2	leucine-rich repeats and immunoglobulin-like domains 2	0	1	0	0

Fkbp1a	FK506 binding protein 1a	0	1	0	0
Chpf	chondroitin polymerizing factor	0	0	1	0
Nlrp4f	NLR family, pyrin domain containing 4F	0	1	0	0
Cdc14b	CDC14 cell division cycle 14B	0	1	0	0
Rnf125	ring finger protein 125	0	1	0	0
Ttll4	tubulin tyrosine ligase-like family, member 4	0	0	0	1
Lpp	LIM domain containing preferred translocation partner in lipoma	0	0	0	1
Map2k4	mitogen-activated protein kinase kinase 4	1	0	0	0
Cacul1	CDK2 associated, cullin domain 1	0	1	0	0
Rbfox2	RNA binding protein, fox-1 homolog (C. elegans) 2	0	1	0	0
Adgrb3	adhesion G protein-coupled receptor B3	1	0	0	0
Rbm4b	RNA binding motif protein 4B	1	0	0	0
Atp7a	ATPase, Cu++ transporting, alpha polypeptide	0	1	0	0
Pds5b	PDS5, regulator of cohesion maintenance, homolog B (S. cerevisiae)	1	0	0	0
Lrrc58	leucine rich repeat containing 58	0	0	1	0
Ogt	O-linked N-acetylglucosamine (GlcNAc) transferase (UDP-N-acetylglucosamine:polypeptide-N-acetylglucosaminyl transferase)	0	1	0	0
Ccnd3	cyclin D3	0	0	1	0
Irf2bpl	interferon regulatory factor 2 binding protein-like	0	1	0	0
Fhod3	formin homology 2 domain containing 3	0	0	0	1
Mtmr3	myotubularin related protein 3	0	1	0	0
Slc41a2	solute carrier family 41, member 2	0	1	0	0
Gpatch8	G patch domain containing 8	0	1	0	0
Ankrd12	ankyrin repeat domain 12	1	0	0	0
Cnot6l	CCR4-NOT transcription complex, subunit 6-like	0	0	1	0
B3galt1	UDP-Gal:betaGlcNAc beta 1,3-galactosyltransferase, polypeptide 1	0	0	1	0
Tet3	tet methylcytosine dioxygenase 3	0	1	0	0
Ttc21b	tetratricopeptide repeat domain 21B	0	1	0	0
Cobll1	Cobl-like 1	0	1	0	0
Sidt2	SID1 transmembrane family, member 2	0	1	0	0
5031414D18Rik	RIKEN cDNA 5031414D18 gene	0	1	0	0
Vat1	vesicle amine transport protein 1 homolog (T californica)	0	1	0	0
Dpp4	dipeptidylpeptidase 4	0	1	0	0
Fbxo33	F-box protein 33	0	0	1	0
Nfkbiz	nuclear factor of kappa light polypeptide gene enhancer in B cells inhibitor, zeta	1	0	0	0
Pdzrn3	PDZ domain containing RING finger 3	1	0	0	0
Tab3	TGF-beta activated kinase 1/MAP3K7 binding protein 3	0	1	0	0
Pcdh17	protocadherin 17	0	1	0	0
Ky	kyphoscoliosis peptidase	0	1	0	0
Fam179b	family with sequence similarity 179, member B	1	0	0	0
Frmpd1	FERM and PDZ domain containing 1	0	1	0	0
Rsf1	remodeling and spacing factor 1	0	1	0	0
Kcnc2	potassium voltage gated channel, Shaw-related subfamily, member 2	0	1	0	0
Rnf38	ring finger protein 38	1	0	0	0
Dync1li2	dynein, cytoplasmic 1 light intermediate chain 2	0	1	0	0
Ythdc1	YTH domain containing 1	0	1	0	0

Cox8a	cytochrome c oxidase subunit VIIIa	1	0	0	0
Slain2	SLAIN motif family, member 2	0	0	0	1
Slc17a2	solute carrier family 17 (sodium phosphate), member 2	0	1	0	0
Kctd1	potassium channel tetramerisation domain containing 1	0	1	0	0
9530068E07Rik	RIKEN cDNA 9530068E07 gene	0	0	0	1
Serbp1	serpine1 mRNA binding protein 1	0	1	0	0
Ppp1r11	protein phosphatase 1, regulatory (inhibitor) subunit 11	0	1	0	0
Pcdh8	protocadherin 8	0	0	0	1
Tmem255a	transmembrane protein 255A	0	1	0	0
Alx1	ALX homeobox 1	0	0	0	1
Ago2	argonaute RISC catalytic subunit 2	0	1	0	0
Cab39	calcium binding protein 39	0	1	0	0
Siah1a	seven in absentia 1A	0	1	0	0
Syde2	synapse defective 1, Rho GTPase, homolog 2 (C. elegans)	0	1	0	0
Ehmt1	euchromatic histone methyltransferase 1	1	0	0	0
Spryd3	SPRY domain containing 3	0	1	0	0
Otud4	OTU domain containing 4	0	1	0	0
D15Ertd621e	DNA segment, Chr 15, ERATO Doi 621, expressed	0	1	0	0
Mycn	v-myc myelocytomatosis viral related oncogene, neuroblastoma derived (avian)	1	0	0	0
Islr	immunoglobulin superfamily containing leucine-rich repeat	0	1	0	0
Ctbp1	C-terminal binding protein 1	1	0	0	0
Atg14	autophagy related 14	0	1	0	0
Tob1	transducer of ErbB-2.1	1	0	0	0
Wdtc1	WD and tetratricopeptide repeats 1	0	1	0	0
Phc3	polyhomeotic-like 3 (Drosophila)	0	1	0	0
Fermt2	fermitin family homolog 2 (Drosophila)	0	1	0	0
Tmem33	transmembrane protein 33	0	1	0	0
Eef1a1	eukaryotic translation elongation factor 1 alpha 1	0	0	0	1
Fam76b	family with sequence similarity 76, member B	1	0	0	0
Ankrd13b	ankyrin repeat domain 13b	0	0	1	0
Bank1	B cell scaffold protein with ankyrin repeats 1	1	0	0	0
Rnf144b	ring finger protein 144B	0	1	0	0
Fkbp14	FK506 binding protein 14	1	0	0	0
Phf20	PHD finger protein 20	0	1	0	0
Cdon	cell adhesion molecule-related/down-regulated by oncogenes	0	1	0	0
Stox2	storkhead box 2	0	1	0	0
Satb2	special AT-rich sequence binding protein 2	0	1	0	0
Higd1a	HIG1 domain family, member 1A	0	1	0	0
Fchsd1	FCH and double SH3 domains 1	0	1	0	0
Nfe2l1	nuclear factor, erythroid derived 2,-like 1	0	0	1	0
Ensa	endosulfine alpha	1	0	0	0
Trps1	trichorhinophalangeal syndrome I (human)	1	0	0	0
Dsel	dermatan sulfate epimerase-like	0	1	0	0
Pbx3	pre B cell leukemia homeobox 3	0	1	0	0
Ptpn3	protein tyrosine phosphatase, non-receptor type 3	0	1	0	0
Smurf1	SMAD specific E3 ubiquitin protein ligase 1	0	1	0	0
Scgb3a2	secretoglobin, family 3A, member 2	1	0	0	0
Kat7	K(lysine) acetyltransferase 7	0	0	0	1
Tasp1	taspase, threonine aspartase 1	0	1	0	0

Rreb1	ras responsive element binding protein 1	0	1	0	0
Tbc1d19	TBC1 domain family, member 19	1	0	0	0
Tmem135	transmembrane protein 135	0	1	0	0
Ypel5	yippee-like 5 (Drosophila)	0	0	0	1
Fam60a	family with sequence similarity 60, member A	0	1	0	0
Magi2	membrane associated guanylate kinase, WW and PDZ domain containing 2	0	0	0	1
Lats1	large tumor suppressor	0	1	0	0
Rab11fip2	RAB11 family interacting protein 2 (class I)	0	1	0	0
Negr1	neuronal growth regulator 1	1	0	0	0
Baz2a	bromodomain adjacent to zinc finger domain, 2A	0	1	0	0
Prrc2c	proline-rich coiled-coil 2C	0	1	0	0
Sema3d	sema domain, immunoglobulin domain (Ig), short basic domain, secreted, (semaphorin) 3D	0	1	0	0
Cdk6	cyclin-dependent kinase 6	0	1	0	0
Ankib1	ankyrin repeat and IBR domain containing 1	0	1	0	0
Gpr63	G protein-coupled receptor 63	0	1	0	0
Wdr47	WD repeat domain 47	0	1	0	0
Abhd13	abhydrolase domain containing 13	0	1	0	0
Arglu1	arginine and glutamate rich 1	1	0	0	0
Zfp609	zinc finger protein 609	0	1	0	0
Btaf1	BTAF1 RNA polymerase II, B-TFIID transcription factor-associated, (Mot1 homolog, S. cerevisiae)	0	1	0	0
Erc2	ELKS/RAB6-interacting/CAST family member 2	0	1	0	0
Fam208a	family with sequence similarity 208, member A	1	0	0	0
Szrd1	SUZ RNA binding domain containing 1	0	1	0	0
Ino80d	INO80 complex subunit D	1	0	0	0
Wwp1	WW domain containing E3 ubiquitin protein ligase 1	0	1	0	0
Pcf11	cleavage and polyadenylation factor subunit homolog (S. cerevisiae)	0	1	0	0
Wapal	wings apart-like homolog (Drosophila)	0	1	0	0
Arhgap32	Rho GTPase activating protein 32	0	1	0	0
Ago1	argonaute RISC catalytic subunit 1	0	1	0	0
Sox5	SRY (sex determining region Y)-box 5	0	0	1	0
Rnf10	ring finger protein 10	0	1	0	0
Ptpre	protein tyrosine phosphatase, receptor type, E	1	0	0	0
Tlk1	tousled-like kinase 1	0	1	0	0
Wdr60	WD repeat domain 60	0	0	0	1
Bhmt2	betaine-homocysteine methyltransferase 2	0	1	0	0
Ppig	peptidyl-prolyl isomerase G (cyclophilin G)	0	1	0	0
Sprr2i	small proline-rich protein 2I	0	0	0	1
Papd4	PAP associated domain containing 4	1	0	0	0
0610010F05Rik	RIKEN cDNA 0610010F05 gene	1	0	0	0
Tmc7	transmembrane channel-like gene family 7	0	1	0	0
Cilp	cartilage intermediate layer protein, nucleotide pyrophosphohydrolase	0	0	0	1
Tmem183a	transmembrane protein 183A	0	1	0	0
Fam63b	family with sequence similarity 63, member B	1	0	0	0
Hoxd1	homeobox D1	0	0	1	0
Reln	reelin	0	1	0	0
Ago4	argonaute RISC catalytic subunit 4	0	1	0	0
Dmtf1	cyclin D binding myb-like transcription factor 1	0	1	0	0

Kcnj12	potassium inwardly-rectifying channel, subfamily J, member 12	1	0	0	0
Ube2q1	ubiquitin-conjugating enzyme E2Q (putative) 1	0	1	0	0
Tfap2d	transcription factor AP-2, delta	0	1	0	0
Rgs8	regulator of G-protein signaling 8	0	1	0	0
Garem	GRB2 associated, regulator of MAPK1	0	1	0	0
Smg7	Smg-7 homolog, nonsense mediated mRNA decay factor (C. elegans)	0	1	0	0
Aqp11	aquaporin 11	0	1	0	0
Rasef	RAS and EF hand domain containing	0	1	0	0
Tmem64	transmembrane protein 64	1	0	0	0
Rab9b	RAB9B, member RAS oncogene family	0	1	0	0
Zc2hc1a	zinc finger, C2HC-type containing 1A	0	1	0	0
Ubxn10	UBX domain protein 10	0	1	0	0
Thrap3	thyroid hormone receptor associated protein 3	1	0	0	0
A830010M20Rik	RIKEN cDNA A830010M20 gene	0	1	0	0
Grsf1	G-rich RNA sequence binding factor 1	0	0	0	1
Bhlha9	basic helix-loop-helix family, member a9	0	1	0	0
Tmem74b	transmembrane protein 74B	0	1	0	0
Zbtb39	zinc finger and BTB domain containing 39	0	1	0	0
Csrnp2	cysteine-serine-rich nuclear protein 2	0	1	0	0
Csrnp3	cysteine-serine-rich nuclear protein 3	1	0	0	0
Ccnjl	cyclin J-like	0	1	0	0
Lsm11	U7 snRNP-specific Sm-like protein LSM11	0	1	0	0
Mylk4	myosin light chain kinase family, member 4	0	1	0	0
2310047M10Rik	RIKEN cDNA 2310047M10 gene	0	1	0	0
Med26	mediator complex subunit 26	0	1	0	0
Rtn4rl1	reticulon 4 receptor-like 1	1	0	0	0
Sowahb	sosondowah ankyrin repeat domain family member B	0	0	1	0
Zfp423	zinc finger protein 423	0	0	1	0
Hnrnph2	heterogeneous nuclear ribonucleoprotein H2	1	0	0	0
Pid1	phosphotyrosine interaction domain containing 1	0	1	0	0
Adrb2	adrenergic receptor, beta 2	0	1	0	0
Zswim3	zinc finger SWIM-type containing 3	0	1	0	0
Slitrk6	SLIT and NTRK-like family, member 6	0	1	0	0
Eif4g1	eukaryotic translation initiation factor 4, gamma 1	0	0	1	0
Onecut2	one cut domain, family member 2	0	1	0	0
Iqub	IQ motif and ubiquitin domain containing	1	0	0	0
Scaf8	SR-related CTD-associated factor 8	1	0	0	0
Ppm1e	protein phosphatase 1E (PP2C domain containing)	0	1	0	0
Scn4b	sodium channel, type IV, beta	0	1	0	0
Tcaim	T cell activation inhibitor, mitochondrial	0	1	0	0
Mtfr1l	mitochondrial fission regulator 1-like	0	1	0	0
Hmga1	high mobility group AT-hook 1	0	1	0	0
Cldn12	claudin 12	0	1	0	0
Atxn1	ataxin 1	0	0	0	1
Cd24a	CD24a antigen	1	0	0	0
Zfp654	zinc finger protein 654	0	1	0	0
Tet1	tet methylcytosine dioxygenase 1	1	0	0	0
Emc6	ER membrane protein complex subunit 6	0	0	1	0
Rnf152	ring finger protein 152	0	1	0	0
Zcchc5	zinc finger, CCHC domain containing 5	0	1	0	0

Cdca4	cell division cycle associated 4	0	1	0	0
Usp14	ubiquitin specific peptidase 14	0	1	0	0
Nat8l	N-acetyltransferase 8-like	0	1	0	0
Pskh1	protein serine kinase H1	0	1	0	0
Bdnf	brain derived neurotrophic factor	0	1	0	0
Tob2	transducer of ERBB2, 2	0	1	0	0
Ccdc6	coiled-coil domain containing 6	0	1	0	0
Dcun1d3	DCN1, defective in cullin neddylation 1, domain containing 3 (S. cerevisiae)	1	0	0	0
Vstm2a	V-set and transmembrane domain containing 2A	0	1	0	0
Rnf24	ring finger protein 24	0	1	0	0
Efna5	ephrin A5	0	0	0	1
Dcaf5	DDB1 and CUL4 associated factor 5	0	1	0	0
Dcaf7	DDB1 and CUL4 associated factor 7	0	1	0	0
Aff4	AF4/FMR2 family, member 4	0	1	0	0
C1ql3	C1q-like 3	0	1	0	0
Bag5	BCL2-associated athanogene 5	0	1	0	0
Rap2c	RAP2C, member of RAS oncogene family	0	1	0	0
Hoxd10	homeobox D10	1	0	0	0
Fam171a1	family with sequence similarity 171, member A1	0	1	0	0
2310061I04Rik	RIKEN cDNA 2310061I04 gene	0	1	0	0
Usp42	ubiquitin specific peptidase 42	0	1	0	0
Rab11fip5	RAB11 family interacting protein 5 (class I)	1	0	0	0
Plekhm3	pleckstrin homology domain containing, family M, member 3	0	0	0	1
Zfp46	zinc finger protein 46	0	1	0	0
Tacstd2	tumor-associated calcium signal transducer 2	0	1	0	0
Dcun1d4	DCN1, defective in cullin neddylation 1, domain containing 4 (S. cerevisiae)	0	1	0	0
Sox6	SRY (sex determining region Y)-box 6	0	1	0	0
5031410I06Rik	RIKEN cDNA 5031410I06 gene	0	1	0	0
Casr	calcium-sensing receptor	0	1	0	0
Btla	B and T lymphocyte associated	0	1	0	0
Sema5b	sema domain, seven thrombospondin repeats (type 1 and type 1-like), transmembrane domain (TM) and short cytoplasmic domain, (semaphorin) 5B	0	1	0	0
Hbb-y	hemoglobin Y, beta-like embryonic chain	1	0	0	0
Trpm3	transient receptor potential cation channel, subfamily M, member 3	0	1	0	0
Tnrc6a	trinucleotide repeat containing 6a	1	0	0	0
Zfp608	zinc finger protein 608	1	0	0	0
Reep1	receptor accessory protein 1	0	1	0	0
Creb5	cAMP responsive element binding protein 5	0	0	1	0
Dspp	dentin sialophosphoprotein	1	0	0	0
Pom121	nuclear pore membrane protein 121	0	1	0	0
Ficd	FIC domain containing	0	1	0	0
Nebl	nebulette	0	1	0	0
Prr14l	proline rich 14-like	0	1	0	0
Vapb	vesicle-associated membrane protein, associated protein B and C	0	1	0	0
Slc8a1	solute carrier family 8 (sodium/calcium exchanger), member 1	1	0	0	0

Klhl18	kelch-like 18	0	1	0	0
Kcmf1	potassium channel modulatory factor 1	0	0	0	1
Pcdh9	protocadherin 9	0	1	0	0
Unc80	unc-80 homolog (C. elegans)	0	1	0	0
Dach1	dachshund 1 (Drosophila)	0	1	0	0
Klhl26	kelch-like 26	0	1	0	0
Nap1l3	nucleosome assembly protein 1-like 3	1	0	0	0
Dennd1b	DENN/MADD domain containing 1B	0	1	0	0
Rlim	ring finger protein, LIM domain interacting	0	1	0	0
Grm7	glutamate receptor, metabotropic 7	0	1	0	0
Setd3	SET domain containing 3	0	1	0	0
Chd6	chromodomain helicase DNA binding protein 6	0	1	0	0
Colq	collagen-like tail subunit (single strand of homotrimer) of asymmetric acetylcholinesterase	0	1	0	0
Sez6l	seizure related 6 homolog like	0	1	0	0
Krtap6-1	keratin associated protein 6-1	0	0	0	1
Arhgap35	Rho GTPase activating protein 35	0	1	0	0
Cryzl1	crystallin, zeta (quinone reductase)-like 1	0	0	0	1
Zfp68	zinc finger protein 68	1	0	0	0
Kl	klotho	0	1	0	0
Fam133b	family with sequence similarity 133, member B	0	1	0	0
Egln2	egl-9 family hypoxia-inducible factor 2	0	1	0	0
Cds2	CDP-diacylglycerol synthase (phosphatidate cytidylyltransferase) 2	0	1	0	0
Kcnc1	potassium voltage gated channel, Shaw-related subfamily, member 1	0	1	0	0
Eda	ectodysplasin-A	0	1	0	0
Zfp422	zinc finger protein 422	1	0	0	0
Atxn7l3	ataxin 7-like 3	0	1	0	0
Tpt1	tumor protein, translationally-controlled 1	0	1	0	0
Pwwp2b	PWWP domain containing 2B	0	1	0	0
Ap2a1	adaptor-related protein complex 2, alpha 1 subunit	0	1	0	0
Chst8	carbohydrate (N-acetylgalactosamine 4-0) sulfotransferase 8	0	1	0	0
Irak2	interleukin-1 receptor-associated kinase 2	0	1	0	0
Dcc	deleted in colorectal carcinoma	0	0	1	0
Fam78b	family with sequence similarity 78, member B	0	1	0	0
Slc9a6	solute carrier family 9 (sodium/hydrogen exchanger), member 6	0	1	0	0
Plekhh1	pleckstrin homology domain containing, family H (with MyTH4 domain) member 1	0	1	0	0
Kcnd2	potassium voltage-gated channel, Shal-related family, member 2	1	0	0	0
Slc4a4	solute carrier family 4 (anion exchanger), member 4	0	1	0	0
Clasrp	CLK4-associating serine/arginine rich protein	0	1	0	0
Nol4l	nucleolar protein 4-like	0	1	0	0
Kalrn	kalirin, RhoGEF kinase	0	1	0	0
Myt1l	myelin transcription factor 1-like	0	1	0	0
Btbd3	BTB (POZ) domain containing 3	1	0	0	0
Cttnbp2nl	CTTNBP2 N-terminal like	1	0	0	0
Tgif2	TGFB-induced factor homeobox 2	0	1	0	0
Trank1	tetratricopeptide repeat and ankyrin repeat containing 1	0	1	0	0

Ncs1	neuronal calcium sensor 1	0	1	0	0
Bbs5	Bardet-Biedl syndrome 5 (human)	1	0	0	0
Scoc	short coiled-coil protein	0	1	0	0
Naa15	N(alpha)-acetyltransferase 15, NatA auxiliary subunit	0	1	0	0
Cyp26b1	cytochrome P450, family 26, subfamily b, polypeptide 1	0	0	1	0
Rnf217	ring finger protein 217	0	1	0	0
Lin28b	lin-28 homolog B (C. elegans)	1	0	0	0
Slc24a3	solute carrier family 24 (sodium/potassium/calcium exchanger), member 3	0	0	1	0
Nlgn1	neuroligin 1	0	1	0	0
Nr6a1	nuclear receptor subfamily 6, group A, member 1	1	0	0	0
Cnnm2	cyclin M2	1	0	0	0
Tank	TRAF family member-associated Nf-kappa B activator	1	0	0	0
Clasp1	CLIP associating protein 1	1	0	0	0
Tacc1	transforming, acidic coiled-coil containing protein 1	0	1	0	0
Nrxn3	neurexin III	0	1	0	0
Capn6	calpain 6	0	1	0	0
Nms	neuromedin S	1	0	0	0
Gm10220	predicted gene 10220	0	1	0	0
Sptbn2	spectrin beta, non-erythrocytic 2	0	1	0	0
Agtr2	angiotensin II receptor, type 2	1	0	0	0
Csde1	cold shock domain containing E1, RNA binding	0	1	0	0
Ddx3y	DEAD (Asp-Glu-Ala-Asp) box polypeptide 3, Y-linked	0	1	0	0
Zbtb10	zinc finger and BTB domain containing 10	0	1	0	0
Gprin1	G protein-regulated inducer of neurite outgrowth 1	0	0	0	1
Marcks	myristoylated alanine rich protein kinase C substrate	1	0	0	0
Ell	elongation factor RNA polymerase II	0	1	0	0
Ccnd1	cyclin D1	0	0	1	0
Hs3st3b1	heparan sulfate (glucosamine) 3-O-sulfotransferase 3B1	0	1	0	0
Mkrn3	makorin, ring finger protein, 3	1	0	0	0
Ccnyl1	cyclin Y-like 1	1	0	0	0
Dnajc25	DnaJ (Hsp40) homolog, subfamily C, member 25	0	1	0	0
Tbpl1	TATA box binding protein-like 1	0	1	0	0
Tut1	terminal uridylyl transferase 1, U6 snRNA-specific	0	0	1	0
Sms	spermine synthase	0	1	0	0
Rybp	RING1 and YY1 binding protein	0	1	0	0
Fam46d	family with sequence similarity 46, member D	0	0	0	1
Gpr174	G protein-coupled receptor 174	0	1	0	0
Zfp449	zinc finger protein 449	0	1	0	0
Pappa2	pappalysin 2	0	1	0	0
Trim68	tripartite motif-containing 68	0	1	0	0
Exoc8	exocyst complex component 8	0	1	0	0
Tnfaip8l3	tumor necrosis factor, alpha-induced protein 8-like 3	0	1	0	0
Kif5a	kinesin family member 5A	0	1	0	0
Zcchc3	zinc finger, CCHC domain containing 3	0	1	0	0
Syndig1	synapse differentiation inducing 1	0	1	0	0
Pdzd8	PDZ domain containing 8	0	1	0	0
Atxn7l3b	ataxin 7-like 3B	0	1	0	0
Plxnc1	plexin C1	0	1	0	0
Arrdc3	arrestin domain containing 3	1	0	0	0
Ranbp6	RAN binding protein 6	0	0	0	1

Fam122a	family with sequence similarity 122, member A	0	0	1	0
Nynrin	NYN domain and retroviral integrase containing	0	1	0	0
Ept1	ethanolaminephosphotransferase 1 (CDP-ethanolamine-specific)	0	1	0	0
Ywhaq	tyrosine 3-monooxygenase/tryptophan 5-monooxygenase activation protein, theta polypeptide	0	1	0	0
Smarcd2	SWI/SNF related, matrix associated, actin dependent regulator of chromatin, subfamily d, member 2	0	1	0	0
Chd2	chromodomain helicase DNA binding protein 2	0	1	0	0
Hoxa3	homeobox A3	0	1	0	0
Seh1l	SEH1-like (S. cerevisiae	0	1	0	0
Plcxd2	phosphatidylinositol-specific phospholipase C, X domain containing 2	0	1	0	0
Tmem170b	transmembrane protein 170B	1	0	0	0
Bcl2l2	BCL2-like 2	0	1	0	0
Slc5a3	solute carrier family 5 (inositol transporters), member 3	0	1	0	0
Lrrc32	leucine rich repeat containing 32	0	1	0	0
Krtap11-1	keratin associated protein 11-1	0	1	0	0
Cecr6	cat eye syndrome chromosome region, candidate 6	0	1	0	0
