# Supplementary material for: Murine trophoblast-derived and pregnancy-associated exosome-enriched extracellular vesicle microRNAs: Implications for placenta driven effects on maternal physiology
Source: PLoS One. 2019 Feb 7;14(2):e0210675. doi: 10.1371/journal.pone.0210675 (PMC6366741; doi:10.1371/journal.pone.0210675)
Supplement: S3 Table — (PDF) [file pone.0210675.s009.pdf]

Table S3. KEGG Pathways Predicted to be Targeted by X-Chromosome Cluster MicroRNAs

KEGG Pathway	Count	%	P-value
Pathways in cancer	38	4.49	2.44E-09
Melanoma	15	1.77	4.99E-07
Focal adhesion	24	2.84	2.64E-06
Wnt signaling pathway	20	2.36	5.21E-06
Axon guidance	18	2.13	1.31E-05
Prostate cancer	14	1.65	4.52E-05
Neurotrophin signaling pathway	16	1.89	1.68E-04
Non-small cell lung cancer	10	1.18	2.27E-04
Pancreatic cancer	11	1.30	4.78E-04
Cell cycle	15	1.77	4.83E-04
Colorectal cancer	12	1.42	5.19E-04
MAPK signaling pathway	23	2.72	7.06E-04
Chronic myeloid leukemia	11	1.30	7.41E-04
Glioma	10	1.18	8.35E-04
Renal cell carcinoma	10	1.18	0.001604307
Small cell lung cancer	11	1.30	0.001780412
Progesterone-mediated oocyte maturation	11	1.30	0.001780412
Endometrial cancer	8	0.95	0.004049041
Hedgehog signaling pathway	8	0.95	0.005013717
Oocyte meiosis	12	1.42	0.005523078
p53 signaling pathway	9	1.06	0.005524583
T cell receptor signaling pathway	12	1.42	0.006696641
Acute myeloid leukemia	8	0.95	0.0067723
ErbB signaling pathway	10	1.18	0.007080809
Long-term depression	9	1.06	0.00714702
Ubiquitin mediated proteolysis	13	1.54	0.00727529
mTOR signaling pathway	7	0.83	0.01897059
Insulin signaling pathway	12	1.42	0.020238377
TGF-beta signaling pathway	9	1.06	0.021088161
Bladder cancer	6	0.71	0.023937777
Thyroid cancer	5	0.59	0.026050405
VEGF signaling pathway	8	0.95	0.029858231
Adherens junction	8	0.95	0.029858231
B cell receptor signaling pathway	8	0.95	0.038047822
Tight junction	11	1.30	0.040569199
